# Mechanistic Insights into Synergistic VFA and NH_3_ Recovery Using a Hybrid BPMED–HFMC System

**DOI:** 10.1021/acs.est.6c05370

**Published:** 2026-09-12

**Authors:** Mohammed Tahmid, Nadia Bianco, Alexa Vitti, Hyuck Joo Choi, Marta C. Hatzell

**Affiliations:** † School of Chemical and Biomolecular Engineering, 115724 Georgia Institute of Technology, 311 Ferst Drive NW, Atlanta, Georgia 30332, United States; ‡ George W. Woodruff School of Mechanical Engineering, Georgia Institute of Technology, 770 Ferst Drive NW, Atlanta, Georgia 30332, United States

**Keywords:** bipolar membrane electrodialysis, hollow fiber membrane contactor, volatile fatty acid recovery, electrochemical separations, anaerobic digestate

## Abstract

Volatile fatty acids (VFAs) and ammonia are valuable intermediate compounds generated during anaerobic digestion and are attractive targets for circular carbon and nitrogen management. Selective separation from ion-rich waste streams remains challenging because of the complexity of the waste-based mixture. Here, we develop a hybrid bipolar membrane electrodialysis–hollow fiber membrane contactor (BPMED–HFMC) system that enables simultaneous recovery and electrochemically controlled fractionation of VFAs while corecovering ammonia without the need for costly external acid or base inputs. In BPMED, electromigration separates charged VFA carboxylates and ammonium. The bipolar membrane (BPM) then generates acid and base in situ, which converts the concentrated VFA carboxylates and ammonium into volatile neutral forms. In parallel, HFMC units then recover VFAs and ammonia as distinct product streams via membrane-mediated gas–liquid transport. We demonstrate that the anion exchange membrane selectivity is in the order acetate > propionate > butyrate > valerate, which correlated strongly with the hydrodynamic radius of the VFAs (*R* ^2^ = 0.96–0.99). During treatment of synthetic anaerobic digestate in the BPMED–HFMC system, acetate selectivity over chloride increased to ∼10 and resulting in a VFA product stream >600 mM (∼71% acetate) at 60 A m^–2^. The specific energy consumption was 0.19–0.21 kWh mol^–1^ (VFA + NH_3_) across current densities of 20–60 A m^–2^, ∼50% lower than an HFMC-only control, with electricity and chemical costs of ∼1.6 cents mol^–1^.

## Graphical abstract

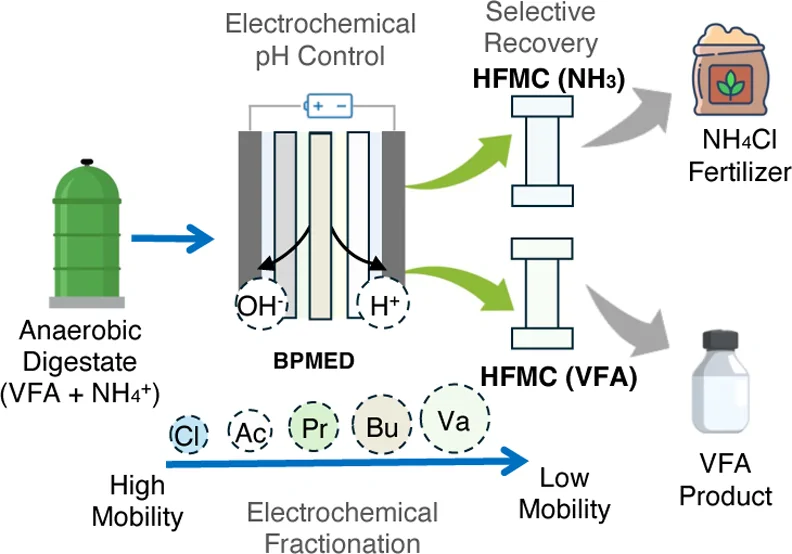

## Introduction

The conversion of organic waste streams, including wastewater sludge, food waste, and animal manure, into valuable chemicals provides a promising pathway toward waste valorization and circular resource management. Anaerobic digestion (AD) is widely deployed to stabilize organic wastes and produce CH_4_-rich biogas.^1,2^ The relatively low economic value of biogas and the additional costs associated with gas cleanup and upgrading often limit deployment without substantial government subsidies.^3,4^ Moreover, the degradation of organic matter during wastewater treatment contributes ∼1.6% of global greenhouse gas (GHG) emissions and ∼5% of global non-CO_2_ emissions^5–7^ highlighting the need for alternative strategies that recover value rather than dissipate embedded chemical energy.

Among the key components of anaerobic digestate, volatile fatty acids (VFAs) and ammonia represent two high-value resources. VFAs are short-chain carboxylic acids with two to six carbon atoms that serve as precursors for higher-value biofuels, biochemicals, and polymer intermediates^8,9^ while ammonia supports global fertilizer production.^10^ VFAs and ammonia coexist in digestate alongside high concentrations of competing inorganic ions (e.g., Cl^–^, Na^+^), neutral solutes, microbes, and suspended solids, creating a fundamental separation challenge that has limited selective recovery and downstream utilization.^11^

Membrane-based separation processes have emerged as promising approaches for VFA recovery from dilute fermentation broths.^12^ Pressure-driven membrane processes such as nanofiltration and reverse osmosis are reported as energy-efficient, with specific energy demands ranging from 0.02–0.20 kWh/kg VFA recovered (0.001–0.01 kWh/mol).^12^ However, the energy requirement is largely associated with hydraulic pressurization of the entire feed stream. As a result, substantial energy is used to transport water rather than selectively recover VFAs, particularly in dilute systems. Pressure-driven membrane processes also exhibit low selectivity for VFAs.^12–14^ In contrast, thermally driven processes such as pervaporation (PV) and membrane distillation (MD) incur energy penalties associated with phase change, with reported specific energies of 3–4 kWh/kg (0.18–0.24 kWh/mol) for PV,^15^ and 4.4–10.9 kWh/kg (0.26–0.66 kWh/mol) for MD.^15,16^ Membrane contactors (MCs) avoid bulk phase change and can selectively transfer VFAs under reactive stripping conditions, but typically require continuous external addition of acids or bases to sustain the chemical driving force^17–19^ shifting energy and cost burdens upstream.

Electrodialysis (ED) provides a fundamentally different separation mechanism, leveraging electric fields to selectively transport charged species at ambient pressure and temperature.^17,20^ Unlike pressure or thermally driven membrane processes, where energy demand is dominated by hydraulic pressurization or latent heat penalties associated with phase change, the energy input in ED is directly coupled to ionic transport. As a result, ED has been demonstrated to efficiently concentrate dissociated VFAs and nutrients from fermented waste streams, achieving concentration factors exceeding 1.5-fold at specific energies of 0.16–3.40 kWh/kg (0.01–0.20 kWh/mol).^21–24^ Under typical anaerobic digestate pH conditions (∼6–7), VFAs (pK_*a*_ ≈ 3.7–4.9) are predominantly present as negatively charged carboxylates,^25^ while ammonia (pK_*a*_ = 9.25) exists as ammonium $\left({\mathrm{NH}}_{4}^{+}\right)$ .^26^ Consequently, both VFAs and ${\mathrm{NH}}_{4}^{+}$ undergo simultaneous electromigration and concentration into one chamber during ED operation. This limits the ability of standalone ED to selectively separate VFAs and ${\mathrm{NH}}_{4}^{+}$ into distinct product streams.

Hollow fiber membrane contactors (HFMCs) offer a complementary separation mechanism by enabling gas–liquid mass transfer under reactive stripping conditions, allowing volatile species to be selectively captured while nonvolatile solutes remain in the aqueous phase.^17^ In digestate systems, HFMCs enable separation based on volatility rather than charge, providing selectivity that is fundamentally inaccessible to ED alone. However, HFMCs cannot achieve high concentration factors from dilute fermentation products^23^ or fractionate charged species in the presence of competing inorganic ions. Alternative membrane-based recovery approaches have also been developed that exploit hydrophobicity rather than volatility as the primary separation mechanism. Preferential partitioning of medium-chain carboxylic acids (MCCAs) into hydrophobic membrane phases has enabled high selectivity relative to short-chain carboxylic acids and other fermentation products through supported liquid membranes, pertraction systems, and hydrophobic polymer membranes.^27–29^ Selective MCCA recovery exceeding 90% has been reported using supported liquid membranes containing hydrophobic extractants,^27^ while pertraction coupled with membrane electrolysis has enabled concentration and phase separation of MCCAs from fermentation broths.^28^ More recently, solvent-free extraction using poly­(dimethylsiloxane) (PDMS) membranes has demonstrated high selectivity for MCCAs over short-chain carboxylic acids, highlighting the potential of hydrophobic partitioning as an alternative membrane-based separation mechanism.^29^

The complementary limitations of ED and HFMC highlight the need for integrated separation strategies that combine electrochemical ion transport with volatility-based separations. To our knowledge, no existing platform simultaneously (i) concentrates dilute fermentation products, (ii) selectively separates VFA and ammonia into chemically distinct streams, as usable products or feedstock for downstream processes, and (iii) operates without reliance on externally supplied acids or bases. Moreover, existing studies largely emphasize system-level performance, with limited mechanistic understanding of how competitive electromigration and diffusive mass transport across membranes would govern product distribution in integrated resource recovery systems.

Here, we develop a hybrid bipolar membrane electrodialysis–hollow fiber membrane contactor (BPMED–HFMC) system that enables simultaneous recovery of ammonia and VFAs into two chemically distinct product streams. In this architecture, BPMED electrochemically separates, concentrates, and then acidifies the VFA carboxylates and basifies the ammonium prior to the HFMC. In the HFMCs, selective recovery is achieved via membrane-mediated gas–liquid mass transfer under reactive stripping conditions, while nonvolatile species are retained in the aqueous phase.^30^ Below we examine mechanism for VFA migration in the ion exchange membranes, and perform a time-resolved flux analysis in the BPMED–HFMC system to demonstrate the temporal evolution of VFA transport and reveal dynamic fractionation behavior during operation. We further determine selectivity coefficients and mass transfer parameters to elucidate the mechanistic origins of VFA fractionation under competitive transport conditions to identify design priorities in hybrid electrochemical–membrane separation systems.

## Materials and Methods

### Chemicals and Synthetic Anaerobic Digestate Preparation

Sodium acetate, sodium propionate, valeric acid, ammonium chloride, sodium chloride, sodium sulfate and all other reagents were ACS grade or higher purity (>99%, Sigma-Aldrich, USA). Sodium butyrate was >98% purity (Sigma-Aldrich, USA). Sodium valerate was prepared by neutralizing valeric acid with NaOH to near-neutral pH prior to use. Deionized (DI) water (18.2 MΩ·cm) was used for all solution preparation. A synthetic anaerobic digestate was prepared to mimic the major composition of VFA-containing fermentation broths, including short-chain VFAs, ammonium, and background inorganic salts. The detailed composition of the synthetic digestate is provided in Table S2 and S3 Supporting Information. All experiments were conducted at room temperature (23 ± 2 °C). A 0.4 M Na_2_SO_4_ solution was used as the electrode rinse solution in all ED experiments.

### Hybrid BPMED–HFMC System Configuration and Operation

Bipolar membrane electrodialysis (BPMED) is a variant of conventional electrodialysis in which a bipolar membrane (BPM) is inserted between each cation exchange membrane (CEM) and anion exchange membrane (AEM), forming a repeating CEM-BPM-AEM unit.^31^ Under an applied electric field, ionic current is carried by ion migration through the membranes and by water splitting at the BPM, producing OH^–^ and H^+^ ions in situ. In practice, BPMED stacks may contain many such repeating units. We conducted batch BPMED experiments using a bench-scale ED 64004 electrodialyzer (PCCell GmbH, Germany) operated in chronopotentiometric mode with a BioLogic VMP3 multichannel potentiostat. The cell consists of a Pt/Ir-MMO–coated titanium anode, a stainless steel cathode, and five repeating units, each with an active membrane area of 8 × 8 cm^2^. Each repeating unit consists of one AEM (PC Acid 60), one CEM (PC SK), and one BPM (PC bip-ED) (all from PCCell GmbH, Germany), separated by polymer spacers.

In the BPMED stack, we circulated synthetic digestate through the central compartment between the AEM and CEM. At the operating feed pH (∼6.3), VFAs (p*K*_*a*_ ≈ 3.75–4.9)^25,32^ are predominantly present as negatively charged carboxylates, while ammonia is present primarily as ammonium ( ${\mathrm{NH}}_{4}^{+}$ ; p*K*_*a*_ = 9.25).^26^ Under an applied electric field, VFA anions migrate through the AEM toward the adjacent acid-generating compartment, while ${\mathrm{NH}}_{4}^{+}$ ions migrate through the CEM toward the base-generating compartment, thereby electrochemically separating VFAs and ${\mathrm{NH}}_{4}^{+}$ (Figure 1, Figure S1). Water dissociation at the BPM generates H^+^ in the acid compartment (between the BPM and AEM) and OH^–^ in the base compartment (between the BPM and CEM). In the acid compartment, VFA anions are protonated to form undissociated VFAs, while in the base compartment, ${\mathrm{NH}}_{4}^{+}$ is deprotonated to form free ammonia (NH_3_). The electrochemically induced pH shift converts the ionic species into volatile neutral forms, enabling downstream recovery in HFMCs.

**Figure 1:**
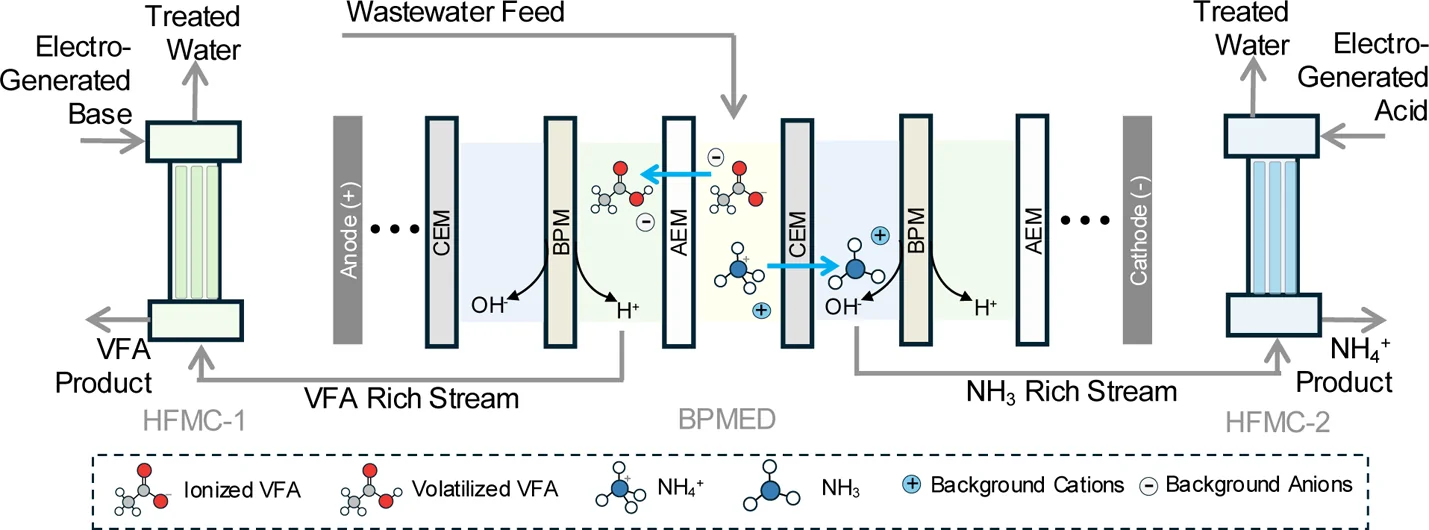
Conceptual schematic of the hybrid BPMED–HFMC system for simultaneous recovery of VFAs and NH_3_ from anaerobic digestate. The BPMED stack comprises multiple repeating CEM–BPM–AEM cell triplets, with the feed distributed across repeating feed compartments of the stack. Ionized VFAs and ${\mathrm{NH}}_{4}^{+}$ in the feed are transported across the AEM and CEM, where they are converted to their volatile neutral forms by the electrochemically generated acid and base. The volatile species are subsequently recovered in two HFMCs, where acidic and basic stripping solutions maintain the driving force for volatility- and pH-shift-driven separation. Background cations (e.g., Na^+^) and anions (e.g., Cl^–^) compete for membrane transport but are not targeted for recovery.

Each acid and base compartment stream from the BPMED cell was directed to a dedicated HFMC. We used two commercial HFMCs (Liqui-Cel EXF-2.5 × 8, 3M, USA) with polypropylene X50 fibers and an effective membrane area of 1.4 m^2^. In each HFMC, BPMED effluent streams were circulated on the shell side, while the stripping solutions were circulated countercurrently through the lumen. Electrochemically generated base (NaOH) and acid (HCl) were produced using a separate BPMED stack operated in conventional mode (as previously described in^33^) with NaCl as the feed electrolyte. Water dissociation at the BPM generated acidic and alkaline streams, which were subsequently collected and used as the HFMC stripping solutions for VFA and NH_3_ recovery. Transmembrane vapor pressure gradients drive diffusion of volatile VFAs and ammonia across the hydrophobic membrane, where they are captured as sodium carboxylates and ammonium chloride in the stripping streams. Nonvolatile species are retained in the aqueous feed, resulting in selective recovery of VFAs and ammonia. The feed circuit was operated in batch mode with 2 L of synthetic digestate. The acid and base loops were initially charged with 250 mL of 10 mM NaCl solution. Electrode compartments were circulated with 300 mL of 0.4 M Na_2_SO_4_ solution. All streams were recirculated using peristaltic pumps (Masterflex, USA) at a flow rate of 10 L h^–1^.

### VFA Transport Experiments in Two-Chamber Electrochemical Cell

We investigated transport selectivity and flux of volatile fatty acids (VFAs), including acetate (Ac^–^, C2), propionate (Pr^–^, C3), butyrate (Bu^–^, C4), and valerate (Va^–^, C5), using a simplified two-chamber ED cell designed to isolate anion transport through an AEM. Chloride (Cl^–^) was included as a representative inorganic competitor due to well-documented transport behavior in ion-exchange membranes^34,35^ and prevalence in VFA-containing fermentation broths and anaerobic digestates.^36,37^ We conducted ED experiments using a bench-scale ED 64002 electrodialyzer (PCCell GmbH, Germany) operated in chronopotentiometric mode with a BioLogic VMP3 multichannel potentiostat. The cell consisted of a Pt/Ir-MMO–coated titanium anode and a stainless-steel cathode separated by a single AEM (PC Acid 60, PCCell GmbH, Germany) with an active membrane area of 8 × 8 cm^2^ (Figure S2). This configuration was analogous to the BPMED cell described above but simplified to a single-membrane system to enable direct measurement of anion transport.

The feed and receiver compartments each contained 500 mL of test solution. Electrode compartments were recirculated with 300 mL of 0.4 M Na_2_SO_4_ as the electrode rinse solution using a peristaltic pump (Masterflex, USA). All experiments were conducted at room temperature (23 ± 2 °C). Equimolar mixtures of sodium acetate, sodium propionate, sodium butyrate, sodium valerate, and sodium chloride were prepared at total concentrations ranging from 5 to 50 mM to span the range of VFA concentrations commonly encountered in fermentation broths and anaerobic digestates. Additional experiments were conducted using fixed VFA concentrations and variable Cl^–^ concentrations to probe competitive transport and flux coupling effects. Anion fluxes were quantified from time-resolved concentration changes in the feed and receiver compartments. Transport selectivity and flux coupling behavior were calculated as described in the Key Performance Metrics and Calculations section. Fluxes and selectivities were calculated from the net concentration change over the 45 min ED experiment. The reported selectivities therefore represent average selectivities over the experimental interval rather than instantaneous values. Previous studies have shown that mixed VFA–chloride systems can exhibit time-dependent transport behavior as solution composition evolves during ED operation.^38^ However, as the feed and receiving volumes were large (500 mL each) and ion removal represented only a fraction of the initial inventory, bulk concentrations remained within a limited range during each experiment, allowing average selectivities to provide a useful measure for comparing relative transport behavior among VFAs.

### Aqueous Chemical Analysis

Ion concentrations were measured using ion chromatography (IC). Cations ( ${\mathrm{NH}}_{4}^{+}$ , Na^+^, and K^+^) were analyzed using a Dionex Aquion ion chromatograph (Thermo Fisher Scientific) equipped with an IonPac CS12A column and a 15 mM methanesulfonic acid eluent. Anions (Cl^–^, ${\mathrm{SO}}_{4}^{2-}$ , and ${\mathrm{PO}}_{4}^{3-}$ ) were measured using an IonPac AS22 column with a carbonate/bicarbonate eluent (4.5 mM Na_2_CO_3_/2.0 mM NaHCO_3_). Prior to IC analysis, samples were acidified to pH 5 using 0.5 M H_2_SO_4_ unless the initial pH was already below 5, at which nearly all ammonia nitrogen was protonated (pH ≪ pK_*a*_ = 9.25) and detectable as ${\mathrm{NH}}_{4}^{+}$ . VFAs (Ac^–^, Pr^–^, Bu^–^, Va^–^) were quantified by high-performance liquid chromatography (HPLC) using a Waters ACQUITY Arc system equipped with a Bio-Rad Aminex HPX-87H column operated at 30 °C. VFAs were detected by UV absorbance at 210 nm using a 5 mM sulfuric acid mobile phase at a flow rate of 0.4 mL min^–1^.

### Key Performance Metrics and Calculations

We quantified VFA transport selectivity between species *A* and *B* using a dimensionless selectivity coefficient, $P_{B}^{A}$ :^39^

1 $$P_{B}^{A}=\frac{t_{A}/C_{A}}{t_{B}/C_{B}}$$

where *t*_*A*_ and *t*_*B*_ are the transport numbers (dimensionless) of species *A* and *B*, respectively, and *C*_*A*_ and *C*_*B*_ (mol L^–1^) are the starting concentrations in the feed compartment. The transport number of a given ionic species *i* expresses the fraction of the total electric current carried by that species under an applied electric field. It is defined in terms of ion fluxes as^40^

2 $$t_{i}=\frac{z_{i}J_{i}}{\underset{j}{\sum }z_{j}J_{j}}$$

where *z*_*i*_ is the ionic charge of species *i*, *J*_*i*_ is molar flux (mol m^–2^ h^–1^), and the summation in the denominator runs over all ionic species *j*.

We calculated the molar flux, *J*_*VFA*_ (mol m^–2^ h^–1^) of VFAs across the IEMs from the change in VFA concentration in the concentrate compartment over each 15 min sampling interval.:

3 $$J_{\mathrm{VFA}}=\frac{\Delta C_{\mathrm{VFA}}V_{\mathrm{feed}}}{NA\Delta t}$$

where Δ*C*_*VFA*_ (mol L^–1^) is the change in VFA concentration over the time interval Δ*t* (h), *V*_feed_ is the feed volume (L), *A* is the active area of a single IEM (m^2^), and *N* is the number of membrane units.

To compare experiments conducted across different operating conditions, we defined a dimensionless time variable:

4 $$\tau =\frac{Q}{V_{\mathrm{feed}}}t$$

where *Q* is the volumetric flow rate (L h^–1^), *V*_feed_ is the feed volume (L), and *t* is time (h). A value of *τ* = 1 corresponds to one residence-time equivalent, representing one complete processing cycle of the feed volume through the system. This normalization enables direct comparison of transport dynamics independent of system scale and hydraulic conditions.

We calculated the mass-transfer coefficient, *k* (m s^–1^) for VFA recovery in the HFMC assuming first-order removal kinetics:^41^

5 $$k_{\mathrm{VFA}}=-\frac{V}{At}\ln\left(\frac{C_{\mathrm{VFA}}\left(t\right)}{C_{\mathrm{VFA}}\left(0\right)}\right)$$

where *V* is the feed volume (m^3^), *A* is the effective membrane area (m^2^), *t* is the contact time (s), and *C*_VFA_(*t*) and *C*_VFA_(0) are the VFA concentrations at time *t* and initially.

We calculated the total specific energy consumption, *SEC*_*total*_ (kWh mol^–1^) for combined VFA and ammonia recovery accounting for both electrical energy input to the BPMED stack and pumping energy associated with fluid transport through the BPMED compartments and the shell and lumen side flow paths of the HFMC, as^42^

6 $${\mathrm{SEC}}_{\mathrm{total}}=\frac{\int_{0}^{t}UI\left(t\right)dt+\underset{j}{\sum }\left(\phi_{j}\Delta P_{j}\right)\Delta t_{\mathrm{total}}}{n_{\mathrm{rec}}}$$

where *U* (V) is the applied voltage, *I*(*t*) (A) is the time-dependent current, *ϕ*_*j*_ (m^3^ s^–1^) and Δ*P*_j_ (Pa) are the volumetric flow rate and pressure drop associated with hydraulic segment *j*, and Δ*t*_total_ is the total operation time (s). The summation index *j* includes all pumped flow paths in the system, namely the BPMED compartments as well as the shell and lumen side flows of the HFMC. Pressure drops were measured experimentally for each hydraulic segment. The recovered moles of product, *n*_rec_, was calculated from the final concentration of VFA and NH_3_ in the product reservoir multiplied by the reservoir volume.

### Statistical Analysis

We evaluated relationships between VFA transport selectivity and physicochemical properties using ordinary least-squares linear regression. The strength of correlations was quantified using the coefficient of determination (*R* ^2^). We assessed differences in mass-transfer coefficients across VFAs and across experimental conditions using one-way analysis of variance (ANOVA). When appropriate, we performed pairwise comparisons using two-tailed Student’s *t*-tests with Bonferroni correction to account for multiple comparisons. Statistical significance was defined at a threshold of *p*  < 0.05 unless otherwise noted. We performed all statistical analyses using Python (SciPy and NumPy libraries). All reported values represent the mean ± standard deviation of replicate experiments.

## Results and Discussion

### VFA Selectivity and Coupled Transport in Electrodialysis

We first quantified VFA selectivity relative to Cl^–^ using a simplified two-chamber ED cell to isolate VFA transport through an AEM. Under equimolar VFA–Cl^–^ feed conditions representative of fermentation broths and anaerobic digestates (5–50 mM), VFA selectivity exhibited a strong dependence on feed concentration (Figure 2a). When each anion was present at 5 mM, selectivity decreased monotonically with increasing VFA chain length, spanning more than an order of magnitude from acetate $\left(P_{{\mathrm{Cl}}^{-}}^{\mathrm{Ace}}=1.19\right)$ to valerate $\left(P_{{\mathrm{Cl}}^{-}}^{\mathrm{Val}}=0.11\right)$ . Increasing the equimolar feed concentration of each ion to 20 and 50 mM reduced selectivity for all VFAs while preserving the same ordering (Ac^–^ > Pr^–^ > Bu^–^ > Va^–^). Ac^–^ selectivity decreased from 1.19 at 5 mM to 0.45 at 20 mM and 0.29 at 50 mM. This attenuation of selectivity with increasing feed concentration is consistent with reported behavior in ED systems for competing monovalent anions^43,44^ including nitrate–chloride separations, where increased ionic strength diminishes apparent selectivity as competitive counterion binding changes with composition of the bulk solution in the feed chamber.^43^

**Figure 2:**
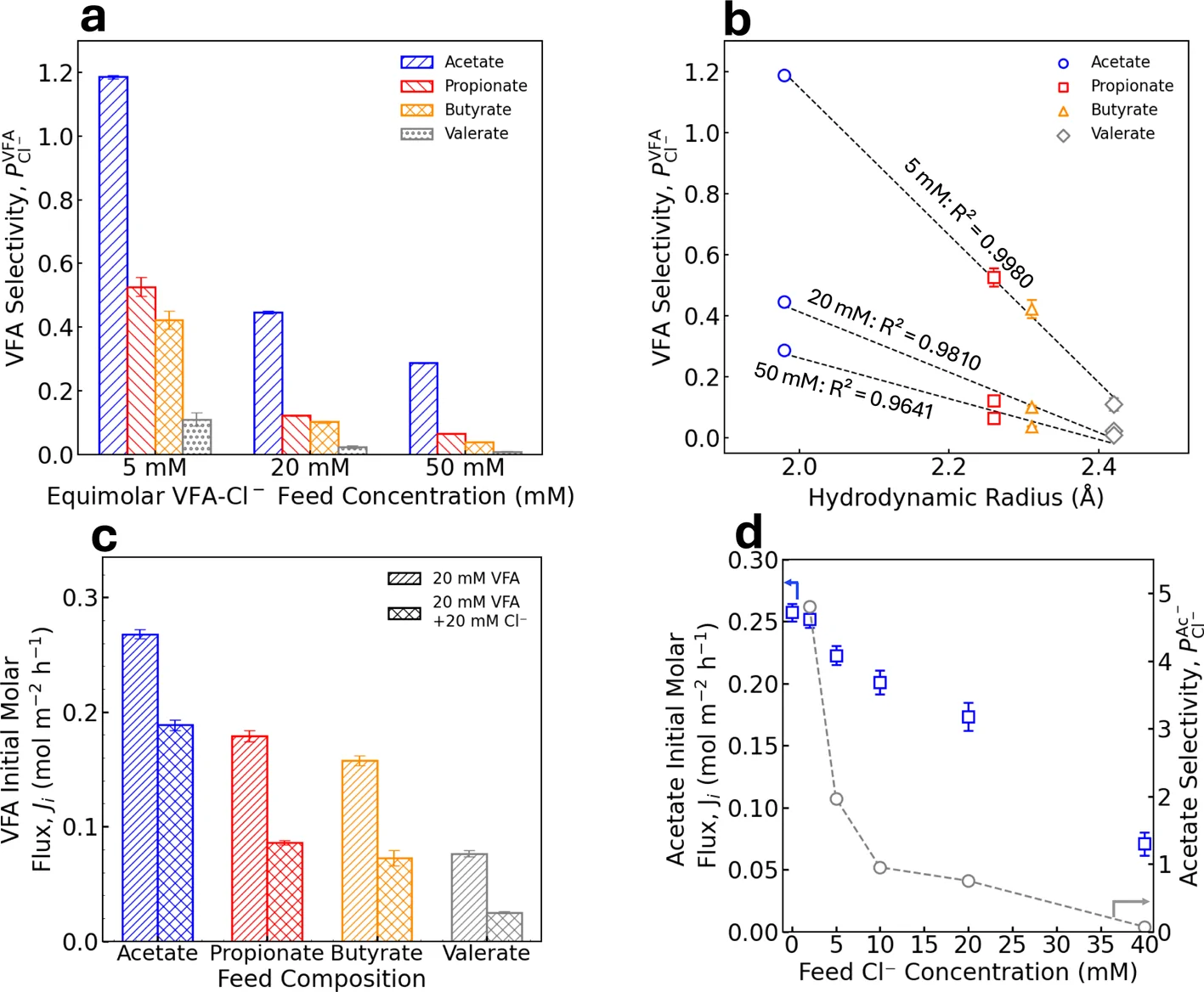
VFA selectivity and coupled transport behavior in two-chamber ED. (a) VFA-to-Cl^–^ selectivity, $P_{{\mathrm{Cl}}^{-}}^{\mathrm{VFA}}$ , for different equimolar feed concentrations (5–50 mM), (b) Linear correlation between VFA selectivity and hydrodynamic radius across all concentrations investigated, (c) Initial VFA molar fluxes measured during the first 15 min of ED operation for single-solute feeds (20 mM VFA) and mixed feeds containing both VFA (20 mM) and Cl^–^ (20 mM), illustrating Cl^–^-induced suppression of VFA transport due to competitive ion migration, (d) Dependence of initial acetate molar flux and selectivity on feed Cl^–^ concentration (0–40 mM), showing progressive flux and selectivity suppression with increasing Cl^–^ content. Selectivity at 0 mM Cl^–^ is theoretically infinite (absence of competing Cl^–^ transport) and is not shown. Error bars represent the standard deviation of triplicate experiments.

Importantly, these experiments were designed to isolate the competitive transport interactions that occur between VFAs and background inorganic anions during migration through an AEM. While chloride remained the dominant charge carrier under all conditions, the relative contribution of individual VFAs to membrane transport differed systematically with VFA chain length and feed composition. These selectivity contrasts provide the physicochemical basis for VFA fractionation in a BPMED–HFMC system. Given the consistent ordering of selectivity across VFAs, we next examined the physicochemical factors governing these differences. We correlated VFA selectivity with hydrodynamic radius, hydration enthalpy, hydrophobicity, and polarizability. Across all concentrations investigated, selectivity exhibited a strong inverse linear relationship with hydrodynamic radius (R^2^ = 0.96–0.99) (Figure 2b), while correlations with other descriptors were substantially weaker (Figure S3 and Table S4). This trend is consistent with size-dependent, hydration-controlled ion mobility, where diffusivity decreases with increasing hydrodynamic radius, as described by the Stokes–Einstein relationship.^11,45^

Beyond selectivity, we observed pronounced coupled transport effects in mixed-anion systems. Initial VFA molar fluxes measured during the first 15 min of ED operation were substantially lower for mixed feeds containing both VFA (20 mM) and Cl^–^ (20 mM) compared to single-solute VFA feeds (20 mM) (Figure 2c). The presence of Cl^–^ suppressed VFA fluxes by approximately 30–55%, with Ac^–^ flux decreasing from ∼2.7 × 10^–1^ to 1.8 × 10^–1^ mol m^–2^ h^–1^ and the magnitude of flux suppression increasing with VFA chain length. This behavior is consistent with competitive ion transport in multicomponent systems, where highly mobile inorganic anions preferentially carry current and suppress the flux of less mobile species. Similar flux attenuation has been reported in ED studies of mixed inorganic anion feeds, including phosphate recovery from urine in the presence of chloride and sulfate.^17,46,47^ In our system, Cl^–^ initially suppresses VFA transport; however, as ED operation proceeds and the feed composition evolves, the degree of Cl^–^-induced flux suppression diminishes, as evidenced by the changing relative fluxes over time (Figure S4). We further probed flux coupling effects by fixing the acetate concentration and varying the Cl^–^ concentration in the feed (Figure 2d). Increasing the feed Cl^–^ concentration from 0 to 40 mM resulted in suppression of Ac^–^ transport, with the initial Ac^–^ molar flux decreasing from 2.6 × 10^–1^ to 7 × 10^–2^ mol m^–2^ h^–1^, corresponding to a ∼70% reduction in flux. Even modest additions of Cl^–^ (2–5 mM) produced measurable decreases in Ac^–^ flux, indicating strong sensitivity of VFA transport to competing inorganic ions. These results demonstrate that VFA fluxes in multicomponent systems cannot be inferred from single-solute transport behavior alone, but instead reflect coupled ion migration and competition among counterions for charge transport through the membrane. Similar behavior was reported in a recent study that shows competing counterions can alter VFA transport properties and selectivities under ED conditions through changes in partitioning and mobility behavior.^48^ Our observations are consistent with these findings and further demonstrate how such competitive transport phenomena influence VFA transport across wastewater-relevant concentration ranges. This behavior directly relates to the BPMED stage, where depletion of highly mobile inorganic ions during operation leads to evolving VFA selectivity and contributes to the time-dependent fractionation behavior observed in the hybrid BPMED–HFMC system in later sections.

### Dynamic Fractionation of VFAs during Hybrid BPMED–HFMC Operation

We next evaluated how the intrinsic VFA selectivity and coupled transport behavior identified above emerge during sequential batch operation of the hybrid BPMED–HFMC system. We treated a synthetic anaerobic digestate representative of fermentation-derived wastewaters, containing mixed VFAs (total VFA ≈ 58 mM) along with Cl^–^, ${\mathrm{NH}}_{4}^{+}$ , and background inorganic ions (Table S3). The temporal evolution of VFA concentrations and flux, expressed as a function of dimensionless time (*τ*), was quantified in the BPMED acid compartment and the downstream HFMC (Figure 3). Progressive accumulation of VFAs occurred in the BPMED acid compartment, where VFA carboxylates transported across the AEM are first concentrated and subsequently protonated by in situ-generated protons at the BPM, enabling volatilization as free acids.

**Figure 3:**
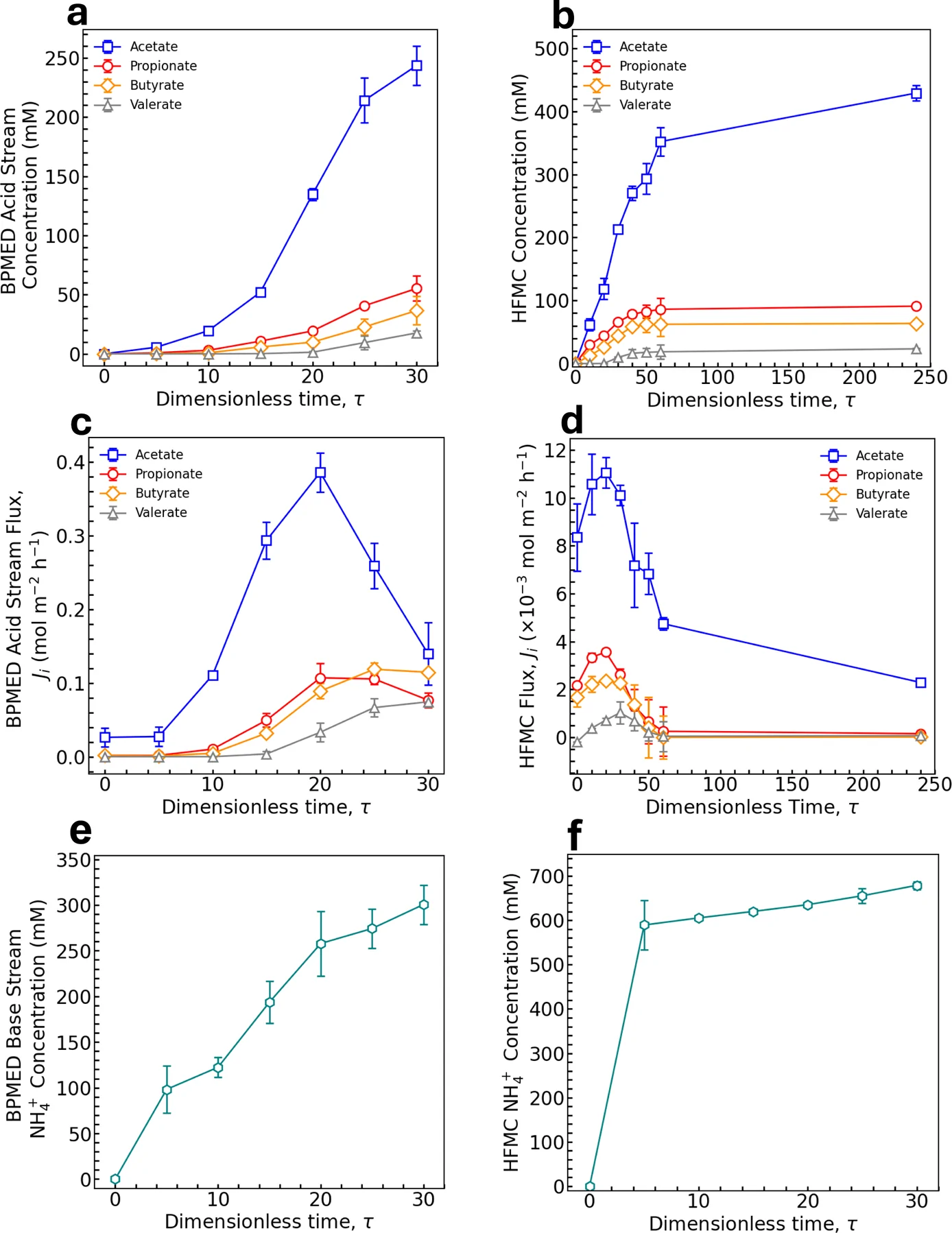
Dynamic concentration and fractionation of VFAs and NH_3_ in hybrid BPMED–HFMC system treating a synthetic anaerobic digestate (current density= 60 A m^–2^) (a) Accumulation of VFAs in the BPMED acid compartment, showing preferential enrichment of acetate relative to longer-chain VFAs, (b) VFA concentrations in the HFMC product stream, demonstrating further concentration, (c) Instantaneous VFA flux (*J_i_*) in the BPMED acid compartment, revealing temporally resolved transport windows governed by competitive ion migration and evolving feed composition, (d) Instantaneous VFA flux in the HFMC that preserves the upstream fractionation, (e) Accumulation of ${\mathrm{NH}}_{4}^{+}$ in the BPMED base compartment operated at a current density of 60 A m^–2^, (f) ${\mathrm{NH}}_{4}^{+}$ concentrations in the HFMC product stream, showing further concentration of ${\mathrm{NH}}_{4}^{+}$ recovered from the BPMED–HFMC system. Error bars denote standard deviations from duplicate experiments.

Although concentrations of all VFAs increased with *τ*, concentration trajectories differed across species. Ac^–^ enriched most rapidly, reaching ∼240 mM by *τ*  ≈ 30, whereas Pr^–^ and Bu^–^ reached ∼55 mM and ∼40 mM, and Va^–^ remained below 20 mM over the same interval (Figure 3a). Separation between VFAs increased with time, indicating progressive amplification of differential transport among species during operation. The normalized concentration factors (*C*/*C*_0_) (Figure S7) further support these observations by accounting for differences in the initial feed composition. Ac^–^ exhibited the greatest enrichment, reaching a concentration factor (*C*/*C*_0_) of ∼6.8 × by *τ* = 30, followed by Pr^–^ (∼6.5×), Va^–^ (∼4.9×), and Bu^–^ (∼4.2×). The divergence in normalized enrichment with increasing *τ* confirms that the observed differences arise from species-dependent transport during BPMED rather than differences in the initial VFA concentrations.

The BPMED acid stream was subsequently processed by the HFMC, where protonated VFAs (acetic acid (HAc, C_2_), propionic acid (HPr, C_3_), butyric acid (HBu, C_4_), and valeric acid (HVa, C_5_)) were selectively transferred across the hydrophobic membrane via volatility-driven diffusion into an electrochemically generated NaOH stripping solution (Table S6), producing sodium–VFA salts. HAc again dominated the HFMC product, exceeding 400 mM by *τ*  ≈ 250, while HPr and HBu plateaued at substantially lower concentrations (∼90 mM and ∼70 mM), and HVa remained below 30 mM (Figure 3b). Enrichment in the HFMC occurred over longer dimensionless times than in the BPMED, consistent with diffusion-limited mass transfer rather than electric-field-driven migration, as has been reported previously for electrochemical stripping systems in which gas transport across a hydrophobic membrane constitutes the rate-limiting step.^49^ This difference of time scales indicates that fractionation is rapidly established in the BPMED, whereas the HFMC governs overall process throughput, highlighting a trade-off between selectivity and productivity in hybrid electrochemical–membrane systems.

The dynamics underlying these concentration profiles were resolved by examining instantaneous fluxes (*J*_*i*_) in the BPMED acid compartment and HFMC (Figure 3c–d). In the BPMED, fluxes (*J*_*i*_) for all VFAs were <3 × 10^–2^ mol m^–2^ h^–1^ at *τ* < 5, consistent with preferential charge transport by more mobile inorganic anions, particularly Cl^–^, during early operation (Figure 3d). As Cl^–^ was progressively removed (Figure S6), VFA transport accelerated, and distinct maxima emerged. Ac^–^ exhibited a pronounced peak in *J*_*i*_ of ∼4 × 10^–1^ mol m^–2^ h^–1^ at *τ* ≈ 15–20, followed by Pr^–^ and Bu^–^ with delayed and lower maxima of ∼(1–1.2) × 10^–1^ mol m^–2^ h^–1^ at *τ* ≈ 20–25, while Va^–^ showed only a modest increase at later *τ*. These staggered maxima define temporally resolved transport windows for each VFA, reflecting differences in mobility and competitive transport under an applied electric field. In the HFMC, flux peaked within a narrower *τ* window and then decayed for all VFAs, with acetate maintaining substantially higher fluxes throughout operation (maximum ∼(0.85–1.15) × 10^–2^ mol m^–2^ h^–1^ at *τ* < 50) (Figure 3d). The absence of strongly separated flux maxima in the HFMC indicates that fractionation is not generated in this step but rather preserved, with the HFMC serving to isolate and concentrate VFAs from the BPMED acid stream while excluding nonvolatile inorganic species. To further assess whether the HFMC contributes to VFA fractionation, we performed experiments using equimolar mixtures of acetate, propionate, butyrate, and valerate at 5, 20, and 50 mM per VFA. Across this concentration range, the overall mass transfer coefficients varied only modestly among VFAs (Figure S10), indicating that HFMC transport exhibits substantially weaker species dependence than the BPMED electromigration step.

Overall, our results show that VFA fractionation in the hybrid BPMED–HFMC system emerges dynamically due to the time-dependent transport of competing ions (e.g., here chloride) which evolve as feed composition changes during operation. Preferential early transport of more mobile VFAs establishes compositional separation in the BPMED, which is subsequently preserved and concentrated by the HFMC. This behavior constitutes an electrochemically driven fractionation process conceptually analogous to staged separations in chromatography or distillation, but governed here by ion migration and volatility rather than phase equilibrium. Ammonia transport and recovery were also quantified during BPMED operation, with ammonia selectively captured via another HFMC using electrochemically generated acid stripping solutions, reaching final ammonium concentrations of 679 mM in the product stream (Figure 3e,f).

### Mechanistic Interpretation of VFA Transport Behavior in the Hybrid BPMED–HFMC System

We resolved the mechanistic origin of dynamic VFA fractionation in the hybrid BPMED–HFMC system using selectivity coefficients and mass transfer parameters (Figure 4). In the BPMED, fractionation arises from differences in ion mobility under an applied electric field, quantified using the VFA-to-Cl^–^ selectivity coefficient, $P_{\mathrm{VFA}/{\mathrm{Cl}}^{-}}$ . Initially, at high inorganic ion concentrations in the feed (initially ∼50 mM Cl^–^, decreasing to ∼15 mM over time), all VFAs exhibit uniformly negligible selectivity $\left(P_{\mathrm{VFA}/{\mathrm{Cl}}^{-}}\approx 0\right)$ , indicating that charge transport is dominated by the more mobile inorganic ions in the feed, particularly Cl^–^ (Figure 4a). As Cl^–^ concentration decreases below ∼10 mM, selectivity increases sharply and divergently among VFAs. Ac^–^ exhibits the largest enhancement, reaching $P_{{\mathrm{Ac}}^{-}/{\mathrm{Cl}}^{-}}∼2$ at Cl^–^ concentrations ∼7 mM, while Pr^–^ and Bu^–^ increase to intermediate values (0.3–0.6), and Va^–^ remains negligible. When Cl^–^ concentrations further decrease to <4 mM, selectivities for the VFAs increase to values as high as 8–40. This evolution in $P_{\mathrm{VFA}/{\mathrm{Cl}}^{-}}$ demonstrates that fractionation is dynamically amplified as the more mobile inorganic ions are depleted. The preserved rank order (Ac^–^ > Pr^–^ > Bu^–^ > Va^–^) indicates systematic differences in VFA transport behavior during competitive electromigration. While the selectivity coefficients reported here reflect the combined effects of membrane partitioning and ion mobility, the observed ordering is consistent with prior studies that identified mobility-related transport differences as important contributors to VFA selectivity in AEMs.^11,48^

**Figure 4:**
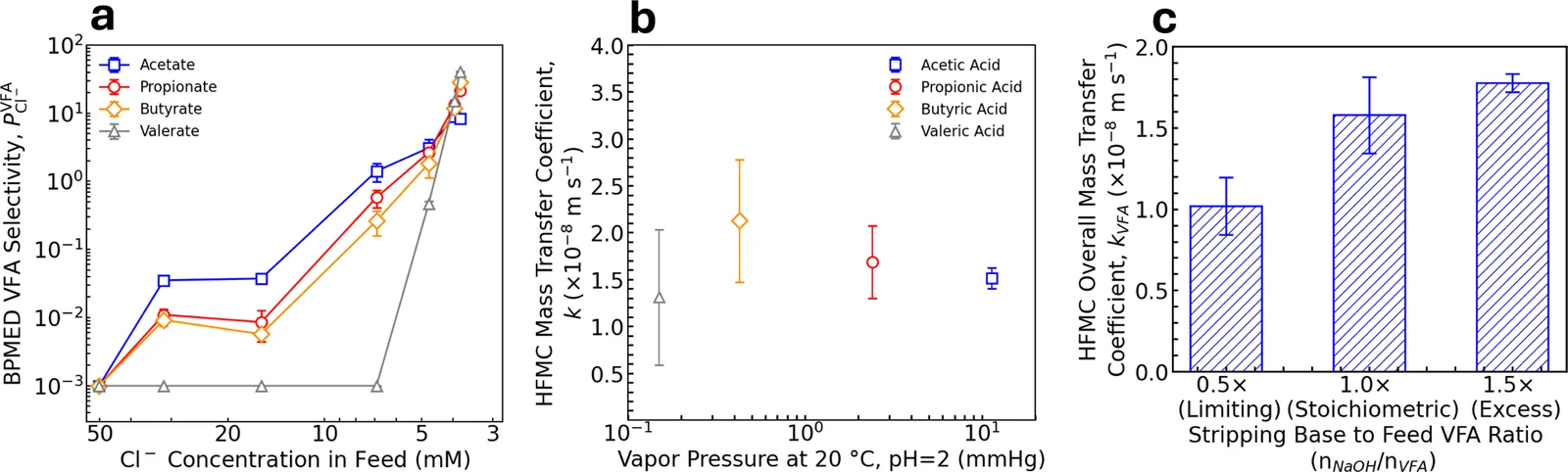
Mechanistic interpretation of transport behavior in the hybrid BPMED–HFMC system. (a) VFA-to-chloride selectivity coefficients $\left(P_{\mathrm{VFA}/{\mathrm{Cl}}^{-}}\right)$ as a function of evolving chloride concentration in the BPMED acid compartment. (b) Overall HFMC mass transfer coefficients (*k*) for individual VFAs under reactive stripping conditions, showing no statistically significant dependence on VFA identity despite large differences in vapor pressure. (c) Effect of NaOH-to-VFA molar ratio on the overall HFMC mass transfer coefficient (*k_VFA_*), indicating transport is dominated by membrane resistance. Error bars represent the standard deviation of duplicate experiments.

Following protonation in the BPMED acid compartment, VFAs are transferred to the HFMC, where transport proceeds via diffusion of undissociated VFAs under reactive stripping conditions. Despite substantial differences in vapor pressure among VFAs, spanning nearly two orders of magnitude from approximately 0.15 mmHg (HVa) to 11.38 mmHg (HAc) at 20 °C (Figure S8), the measured HFMC mass transfer coefficients fall within a narrow range of (1.3–2.1) × 10^–8^ m s^–1^ (Figure 4b). One-way ANOVA confirms that differences in *k* among VFAs are not statistically significant (*p* = 0.74 > 0.05), and pairwise comparisons using two-tailed Student’s *t*-tests with Bonferroni correction likewise reveal no significant differences between any VFA pairs. These results indicate that HFMC transport is not volatility-limited under the conditions employed. Consistent with this interpretation, increasing the NaOH stripping solution-to-VFA molar ratio from limiting (0.5×) to stoichiometric (1.0×) and excess (1.5×) conditions produces only modest increases in the overall HFMC mass transfer coefficient, from 1.0 × 10^–8^ to 1.8 × 10^–8^ m s^–1^ (Figure 4c). We observe a monotonic trend in the HFMC mass transfer coefficient, but the effect is not statistically significant (one-way ANOVA, *p* = 0.11 > 0.05; no significant pairwise differences by Bonferroni-corrected *t*-tests). These observations (Figure 4b–c) indicate that HFMC operation occurs in a mass-transfer-limited regime dominated by membrane-associated resistances rather than gas-phase transport or chemical driving force. Similar membrane resistance–limited behavior has been reported previously for gas diffusion in HFMCs.^50,51^

These mechanistic insights suggest clear design priorities for hybrid electrochemical fractionation systems targeting mixed VFAs. First, fractionation performance is primarily governed by competitive electromigration against background inorganic ions, emphasizing the importance of IEM transport properties that accentuate differences in effective ion mobility among VFAs. Second, as downstream HFMC performance is limited by membrane-associated mass transfer resistances under reactive stripping conditions, hollow fiber membrane design should prioritize optimization of membrane morphology, hydrodynamics, and interfacial transport processes to enhance separation efficiency.

### Energy Consumption, Product Distribution, and Process Implications

The specific energy consumption (SEC), normalized per mole of total recovered VFA + NH_3_ varied from 0.18 to 0.21 kWh mol^–1^ across current densities of 20–60 A m^–2^. Electrical power supplied to the BPMED accounts for the dominant share of the SEC, contributing 75–85% of the total energy input (0.14–0.16 kWh mol^–1^), while pumping energy for the BPMED and HFMC combined contributes <10% (<0.02 kWh mol^–1^). The remaining energy demand arises from the external BPMED unit used to electrochemically generate the HCl and NaOH stripping solutions required for HFMC operation. This acid/base generation step contributes ∼10–20% of the total SEC and decreases slightly with increasing current density. In contrast, HFMC-only operation requires a substantially higher equivalent specific energy input (≈0.40 kWh mol^–1^), arising almost entirely from externally supplied chemicals rather than direct electrical consumption. This value was estimated based on industrial benchmarks for chemical production, with purchased base accounting for approximately 70% of the total SEC and purchased acid contributing the remaining ∼30%. This contrast highlights the key advantage of BPMED–HFMC integration: electrochemical generation of acid and base upstream completely displaces external chemical inputs, enabling internal pH control and reducing overall energy demand (Figure 5a).

**Figure 5:**
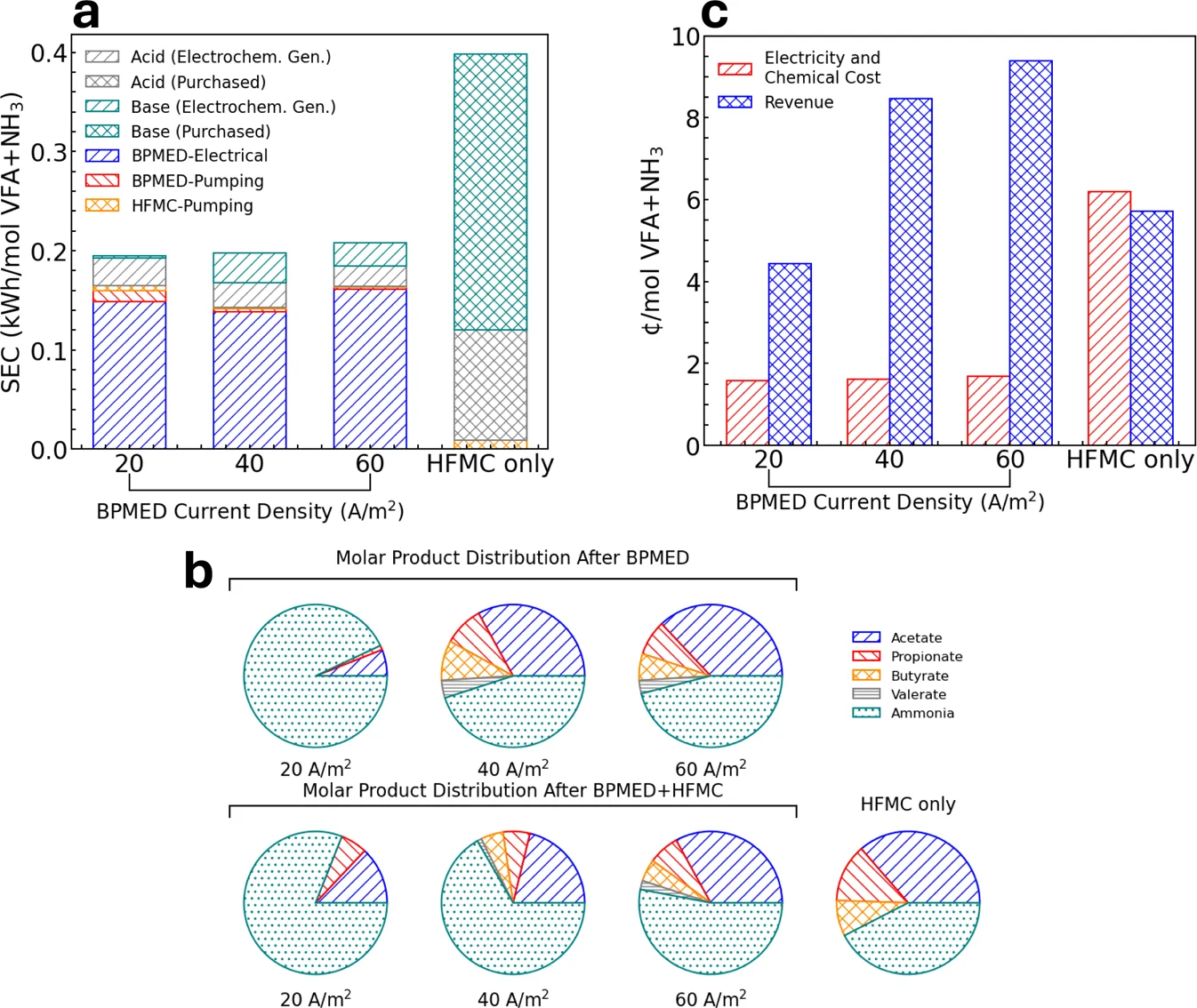
Energy and economic performance of the hybrid BPMED–HFMC system. (a) Breakdown of specific energy consumption for BPMED–HFMC and HFMC-only operation, highlighting the reduction in externally supplied chemicals enabled by electrochemical generation in BPMED. Energy for producing the externally supplied chemicals for HFMC-only operation are estimated using literature-reported industrial benchmarks for NaOH and HCl production.^52,53^ (b) Molar product distribution after BPMED and after BPMED–HFMC operation at different current densities, illustrating current-dependent control over ammonia and VFA recovery. (c) Electricity and chemical cost vs. product revenue for different current densities, demonstrating improved economic performance with increasing current density due to enhanced recovery and favorable shifts in product distribution.

The hybrid BPMED–HFMC configuration enables simultaneous recovery of ammonia and VFAs while allowing active control over their relative distribution in the product streams. After BPMED operation, the recovered molar output varies strongly with the applied current density: ammonia dominates at low current density (≈93% at 20 A m^–2^), whereas VFAs comprise a majority of the recovered products at higher current densities (≈54–55% at 40–60 A m^–2^) (Figure 5b). This shift reflects enhanced electromigration of VFAs as highly mobile inorganic anionic competitors are progressively depleted under stronger electric fields, resulting in increased VFA recovery. Downstream HFMC treatment selectively strips ammonia into an acid stream while stripping VFAs into a base stream, yielding a VFA-enriched product whose composition is likewise tunable with BPMED operating conditions. Following BPMED–HFMC operation, VFAs account for 19%, 34%, and 47% of the recovered molar output at 20, 40, and 60 A m^–2^, while ammonia is concurrently recovered as a distinct product. Across all conditions, HAc remains the dominant VFA (≈50–71% of total VFAs), followed by HPr and HBu, with HVa contributing <5%. These trends demonstrate that current density and residence time provide effective levers for tailoring molar product distributions, enabling flexible process operation to meet downstream utilization or market demands. The overall recovery of VFAs (Table S7) increased with current density. At 60 A/m^2^, recoveries reached 92%, 88%, and 86% for HAc, HPr, and HBu, whereas HVa recovery remained lower (49%), consistent with lower selectivity of valerate (Figure 2).

These energetic and compositional effects translate directly into improved process economics. Electricity and chemical costs for the hybrid BPMED–HFMC system range from 1.58 to 1.69 ¢ mol^–1^ of recovered VFA + NH_3_ at current densities between 20 and 60 A m^–2^ (Figure 5c). Over the same range, product revenue increases substantially, from 4.43 to 9.39 ¢ mol^–1^, driven by increased total recovery and a shift in product distribution toward higher-value VFAs relative to ammonia. Consequently, net value generation, defined here as product revenue minus electricity and chemical cost, increases with BPMED current density. In comparison, HFMC-only operation exhibits a substantially higher electricity and chemical cost (6.20 ¢ mol^–1^) with only moderate product revenue (5.72 ¢ mol^–1^), resulting in limited net value generation. Assumptions used in the energy and economic analysis, including electricity price, acid and base production costs, and the energies associated with their production, are summarized in the Supporting Information Table S9. The economic analysis presented herein focuses on electricity and chemical costs and is intended to facilitate comparison across operating conditions. Capital costs associated with BPMED stacks, IEMs, HFMC modules, and auxiliary equipment were not considered. A comprehensive techno-economic assessment incorporating capital expenditures, membrane replacement schedules, equipment lifetimes, financing assumptions, and process scale effects would be required to assess overall process economics and competitiveness.

### Implications for Wastewater Resource Recovery

We highlight the potential of a hybrid BPMED–HFMC system to reshape how wastewater is managed, shifting treatment objectives from contaminant removal toward selective recovery and valorization of nutrients and organic carbon. We demonstrate simultaneous recovery of ammonia and VFAs with tunable control over product distribution through electrochemical operating parameters. Importantly, the value of this tunability extends beyond maximizing overall recovery. Depending on downstream application requirements, process operation can be directed toward broad recovery of mixed VFA streams or toward enrichment of specific VFA fractions through controlled manipulation of electromigration-driven transport. In our work, preferential enrichment of shorter-chain VFAs emerged naturally during operation, illustrating how electrochemical control can be used to influence product composition without additional separation steps. We also uncovered that this control emerges from intrinsic transport mechanisms, including electromigration, ion competition, and diffusion-limited membrane contactor behavior that governs recovery rates, selectivity, and energy efficiency, rather than relying on chemical additives or complex downstream separations. By adjusting current density and residence time, the hybrid BPMED–HFMC platform enables resource recovery to be steered toward ammonia-rich fertilizers, VFA-rich product streams, or any intermediate ratios. This flexibility provides a strategy for wastewater resource recovery that can adapt to variable feed composition, scale, and local valorization pathways, particularly in decentralized waste treatment contexts.

The VFA product stream recovered in our study remains a mixture of individual VFAs, although the composition can be shifted through electrochemical operating conditions. Such mixed VFA streams are valuable intermediates for a range of biological and chemical conversion pathways, including chain elongation, biopolymer production, and sustainable fuel synthesis.^7^ For applications requiring purified individual VFAs, the concentrated product stream produced by the BPMED–HFMC platform could serve as an upstream enrichment step prior to downstream fractionation technologies such as extraction, adsorption, distillation, membrane separations, or additional electrochemical separation stages.

Realizing the hybrid BPMED–HFMC platform at scale will require advances that reinforce transport-governed separation. Greater selectivity and durability of IEMs under mixed organic–inorganic conditions are needed to sustain electromigration driven fractionation at lower cost, while optimization of hollow fiber membrane transport properties will be critical to overcome mass transfer limitations and enhance recovery efficiency. Real anaerobic digestates also exhibit substantial variability in background ion composition, including bicarbonate/carbonate, sulfate, phosphate, and chloride. These species may compete with VFAs for transport through the IEMs and influence current efficiency, product distribution, and recovery performance. Future studies evaluating the BPMED–HFMC platform using real digestates will be important for assessing system robustness and validating the transport mechanisms identified herein in more complex feed matrices. Furthermore, as BPMED and membrane technologies continue to mature and expand in industrial implementation, advances in membrane performance, stack design, and manufacturing scale may further improve the economic prospects of integrated electrochemical separation platforms.

From a process standpoint, integration with upstream fermentation or anaerobic digestion could further enhance VFA availability, while coupling recovered ammonia and VFAs to downstream synthesis or formulation steps could enable higher-value products beyond direct nutrient recovery, including tailored fertilizer formulations, sustainable aviation fuel intermediates derived from VFAs, and biopolymer precursors. The hybrid BPMED–HFMC framework can also be integrated with renewable electricity and modular deployment strategies, positioning it for incorporation into emerging circular water infrastructure. Together, our findings demonstrate a broader shift from static, energy-intensive wastewater treatment toward an adaptive, electrically driven resource recovery platform that valorize nitrogen and carbon in response to environmental and economic goals.

## Supplementary Material



## Notes

Composition and recipe of synthetic anaerobic digestate; ED setup for VFA transport experiments; relationships between VFA selectivity and physicochemical properties; assumptions used for energy and economic analysis; BPMED voltage and pH data; ammonia concentration profiles; first-order kinetic fits for VFA transport in the HFMC; recovered product stream compositions and recoveries (PDF)
