# Supplementary material for: Mechanistic Insights into Synergistic VFA and NH3 Recovery Using a Hybrid BPMED–HFMC System
Source: Environ Sci Technol. 2026 Sep 12;60(37):26766–76. doi: 10.1021/acs.est.6c05370 (PMC13614185; doi:10.1021/acs.est.6c05370)
Supplement: Supplementary file 1 [file es6c05370_si_001.pdf]

Supporting Information: Mechanistic Insights into Synergistic VFA and NH_3 Recovery Using a Hybrid BPMED–HFMC System

Mohammed Tahmid,[†] Nadia Bianco,[†] Alexa Vitti,[†] Hyuck Joo Choi,[†] and Marta C. Hatzell[*,‡,†]

†*School of Chemical and Biomolecular Engineering, Georgia Institute of Technology, 311 Ferst Drive NW, Atlanta, GA, 30332, USA*

‡*George W. Woodruff School of Mechanical Engineering, Georgia Institute of Technology, 770 Ferst Drive NW, Atlanta, GA, 30332, USA*

E-mail: marta.hatzell@me.gatech.edu

This Supporting Information contains:

Total number of pages: 12

Total number of Figures: 10

Total number of Tables: 9

Table S1: Common VFAs: chemical formulas and major end-use sectors

**VFA**	**Chemical Formula**	**Major End Uses**	**References**
Acetic acid	$C_2H_4O_2$	Production of polymers, adhesives, and dyes; food additive (vinegar); precursor for chemical synthesis.	[1]
Propionic acid	$C_3H_6O_2$	Food preservative and additive; flavoring agent; pharmaceutical applications; animal feed additive.	[1]
Butyric acid	$C_4H_8O_2$	Additive in animal and human food; intermediate in chemical synthesis; solvent; flavoring agent.	[1]
Valeric acid	$C_5H_{10}O_2$	Production of perfumes and cosmetics; food additives through ester derivatives with fruity aromas; plasticizers; pharmaceutical applications.	[2]

Table S2: Composition of the synthetic anaerobic digestate used. Concentrations are reported as mass added per liter of DI water along with the corresponding molar concentration (adapted from[3]).

Compound	Concentration (g L^{-1})	Molar Mass (g mol^{-1})	Concentration (mmol L^{-1})
Sodium acetate	2.78	82.03	33.89
Sodium propionate	0.985	96.06	10.25
Sodium butyrate	0.948	110.10	8.61
Valeric acid	0.532	102.13	4.90
Ammonium chloride	3.21	53.49	60.01
Potassium dihydrogen phosphate	0.05	136.09	0.37
Sodium bicarbonate	0.10	84.01	1.19
Calcium chloride	0.02	110.98	0.18
Magnesium sulfate heptahydrate	0.015	246.48	0.06

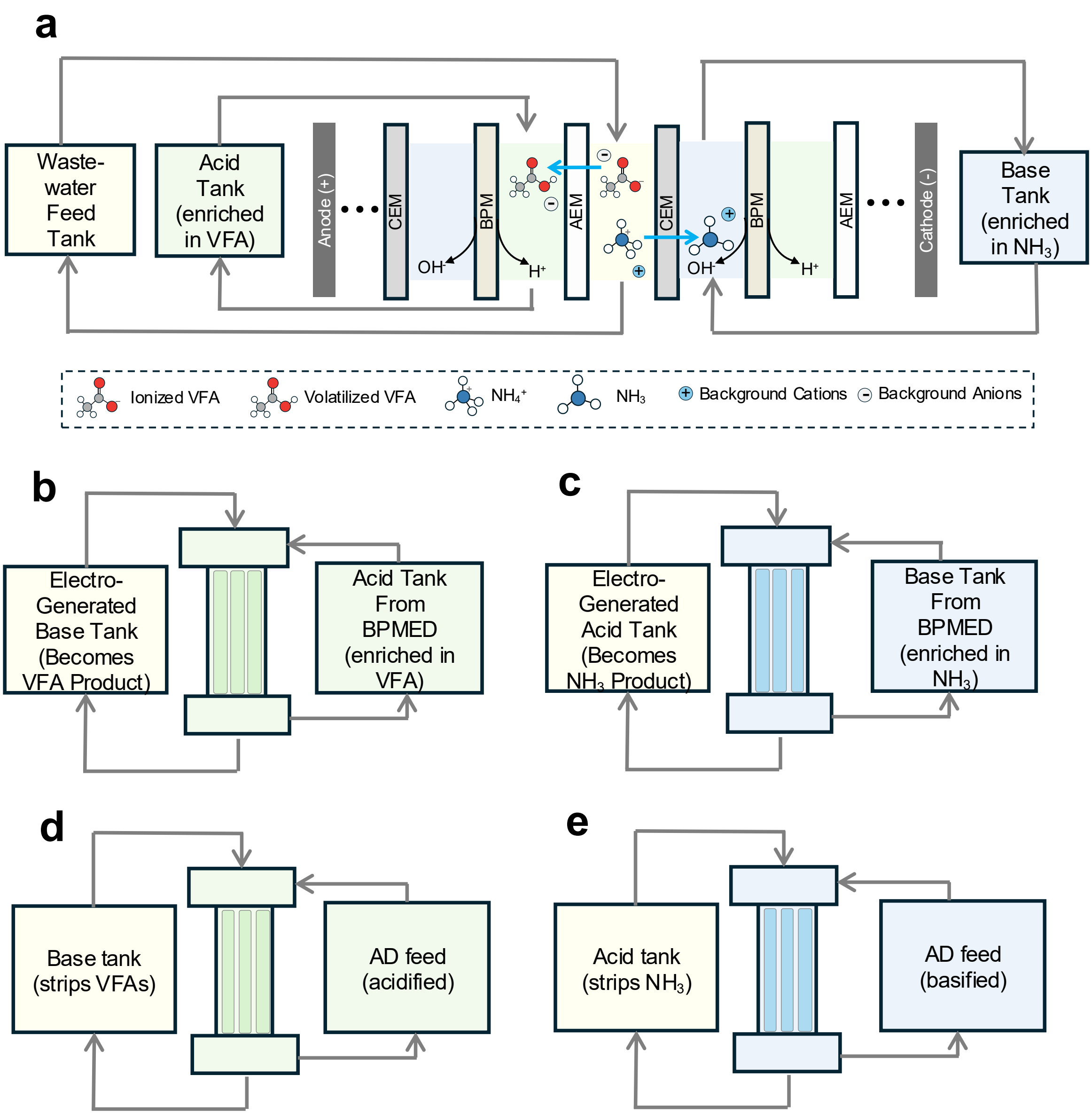

Figure S1: Detailed schematics of the experimental setup. (a) BPMED system showing the feed, acid and base tanks and their connections to the BPMED stack. (b) HFMC configuration used for VFA recovery, illustrating the feed and stripping solution reservoirs and recirculation loops. (c) HFMC configuration used for ammonia recovery, showing the corresponding feed and stripping solution reservoirs and recirculation loops. The VFA and ammonia stripping HFMCs are run in parallel. (d) HFMC-only operation for VFA recovery from anaerobic digestate acidified with externally added HCl (not BPMED-generated acid), using an externally supplied NaOH stripping solution. (e) HFMC-only operation for NH_3 recovery from anaerobic digestate basified with externally added NaOH (not BPMED-generated base), using an externally supplied HCl stripping solution.

Table S3: Calculated aqueous species composition of the synthetic anaerobic digestate derived from the recipe shown in Table S1. Concentrations are reported in mmol L^{-1} and ppm (mg L^{-1}).

Species	Concentration (mmol L^{-1})	Molar Mass (g mol^{-1})	Concentration (ppm)
Na^+	53.94	22.99	1240.19
CH_3COO^-	33.89	59.04	2000.87
$C_2H_5COO^-$	10.25	73.08	749.36
$C_3H_7COO^-$	8.61	87.11	750.05
C_4H_9COOH	4.90	102.13	500.00
NH_4^+	60.01	18.04	1082.60
Cl^-	60.01	35.45	2129.23
K^+	0.37	39.10	14.46
$H_2PO_4^-$	0.37	96.99	35.91
HCO_3^-	1.19	61.02	72.61
Ca^{2+}	0.18	40.08	7.21
Mg^{2+}	0.06	24.31	1.46
SO_4^{2-}	0.06	96.06	5.76

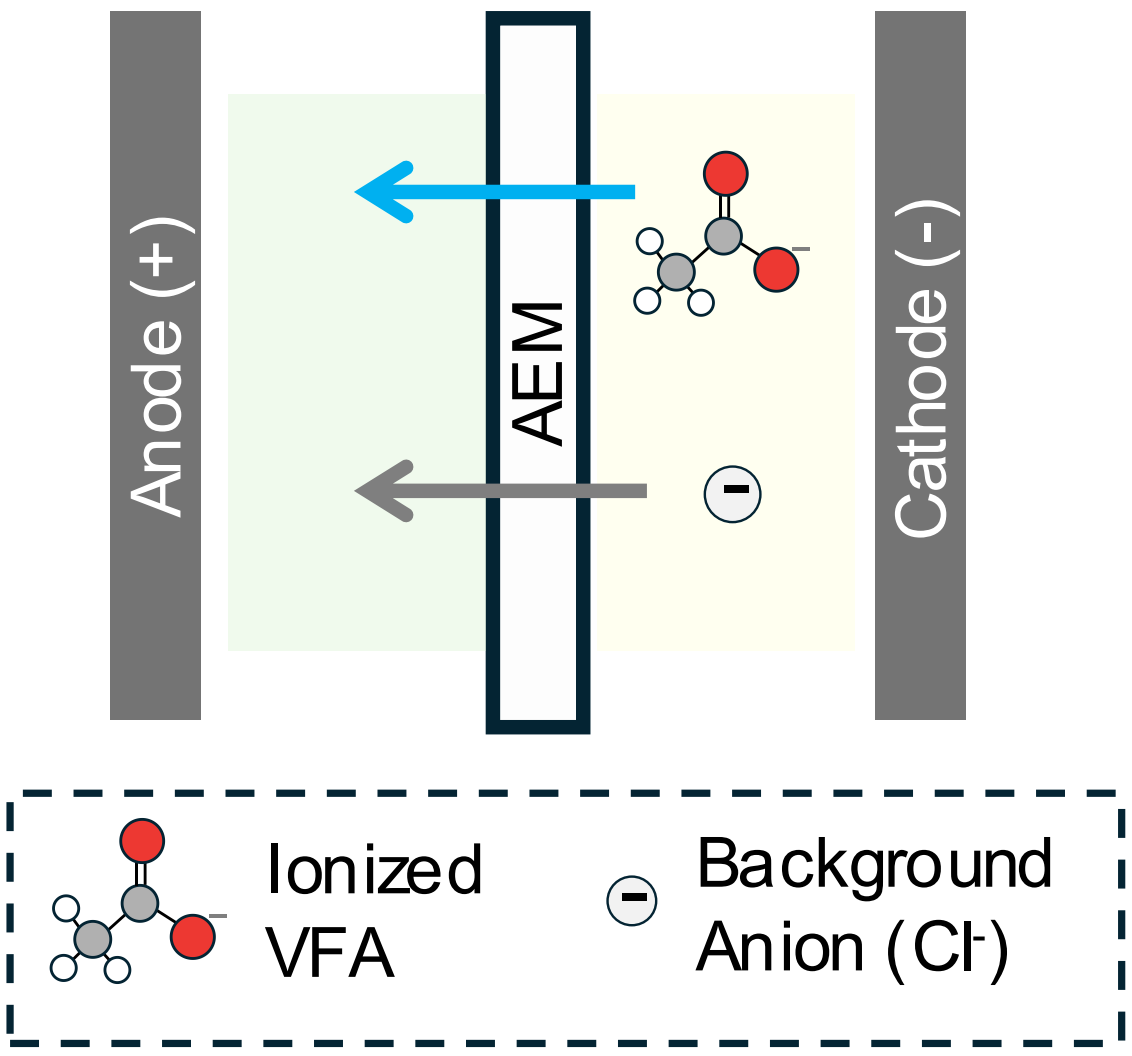

Figure S2: Simplified two-chamber electrodialysis cell designed to isolate anion transport through an AEM

Table S4: Coefficients of determination (R^2) for linear regressions between VFA selectivity and physicochemical descriptors shown in Figure S3, at different equimolar VFA-Cl^- concentrations

Property	R^2 5 mM	R^2 20 mM	R^2 50 mM
Hydration enthalpy (ΔH_{hyd})	0.77	0.62	0.59
Hydrophobicity ($\log K_{ow}$)	0.89	0.77	0.74
Polarizability	0.90	0.79	0.77

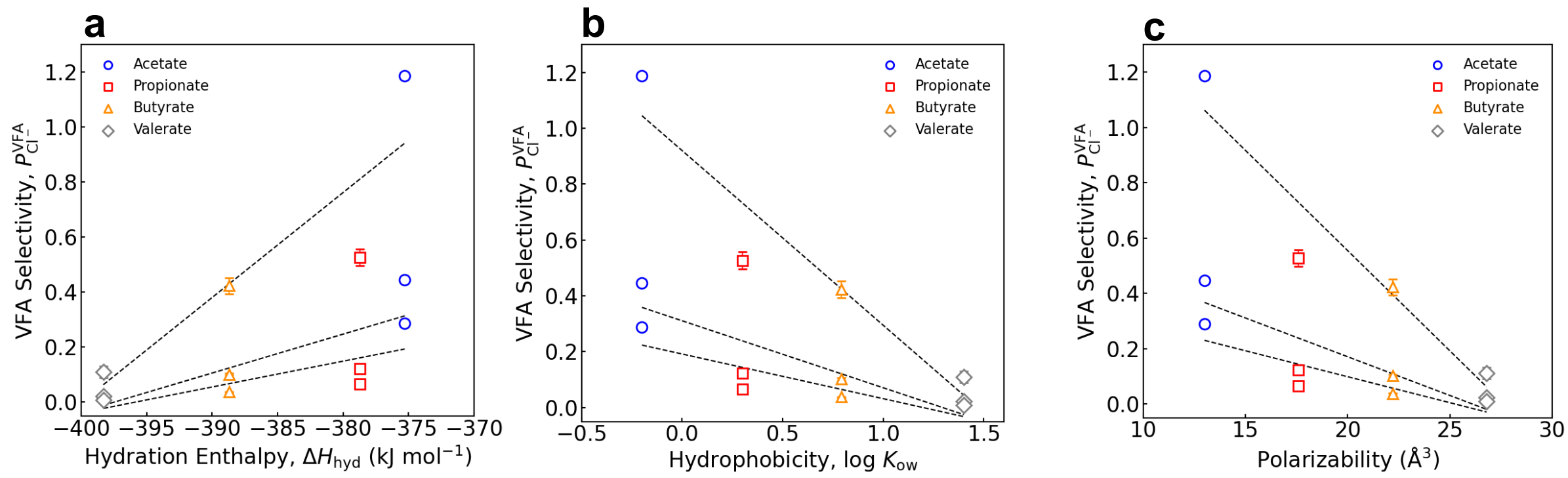

Figure S3: Relationships between VFA selectivity and physicochemical properties of short-chain carboxylates. VFA selectivity $\left(P_{Cl^-}^{VFA}\right)$ for acetate, propionate, butyrate, and valerate is plotted as a function of (a) hydration enthalpy (ΔH_{hyd}), (b) hydrophobicity expressed as the octanol–water partition coefficient ($\log K_{ow}$), and (c) molecular polarizability. Feed solutions contained equimolar concentrations of each of the VFA and Cl^- (5, 20, and 50 mM each). Symbols represent mean values and error bars denote standard deviations from triplicate experiments. Dashed lines indicate linear regressions for each concentration condition. Corresponding coefficients of determination (R^2) are provided in Table S4.

Table S5: pH variation of acid (VFA-rich) and base (NH_3-rich) streams in the BPMED cell at different current densities and operating times.

Current Density (A/m^2)	Stream	Time (h)						
		0	1	2	3	4	5	6
20	Acid stream (VFA rich)	7.6	2.0	1.7	1.6	1.6	1.7	1.7
	Base stream (NH_3 rich)	7.6	11.7	11.7	11.3	10.9	10.7	10.4
40	Acid stream (VFA rich)	7.6	1.3	1.3	1.3	1.6	1.9	2.1
	Base stream (NH_3 rich)	7.6	12.3	12.5	12.6	12.7	12.9	12.9
60	Acid stream (VFA rich)	7.6	1.6	1.3	1.4	2.0	2.0	1.9
	Base stream (NH_3 rich)	7.6	12.8	12.9	12.9	12.6	12.8	12.2

Table S6: Concentrations of electrochemically generated stripping solutions used for ammonia and VFA recovery at different BPMED current densities.

**Current density** (A m^{-2})	**HCl concentration used for NH_3 stripping** (M)	**NaOH concentration used for VFA stripping** (M)
20	0.44	0.04
40	0.80	0.99
60	1.20	1.41
HFMC-only control	0.06	0.23

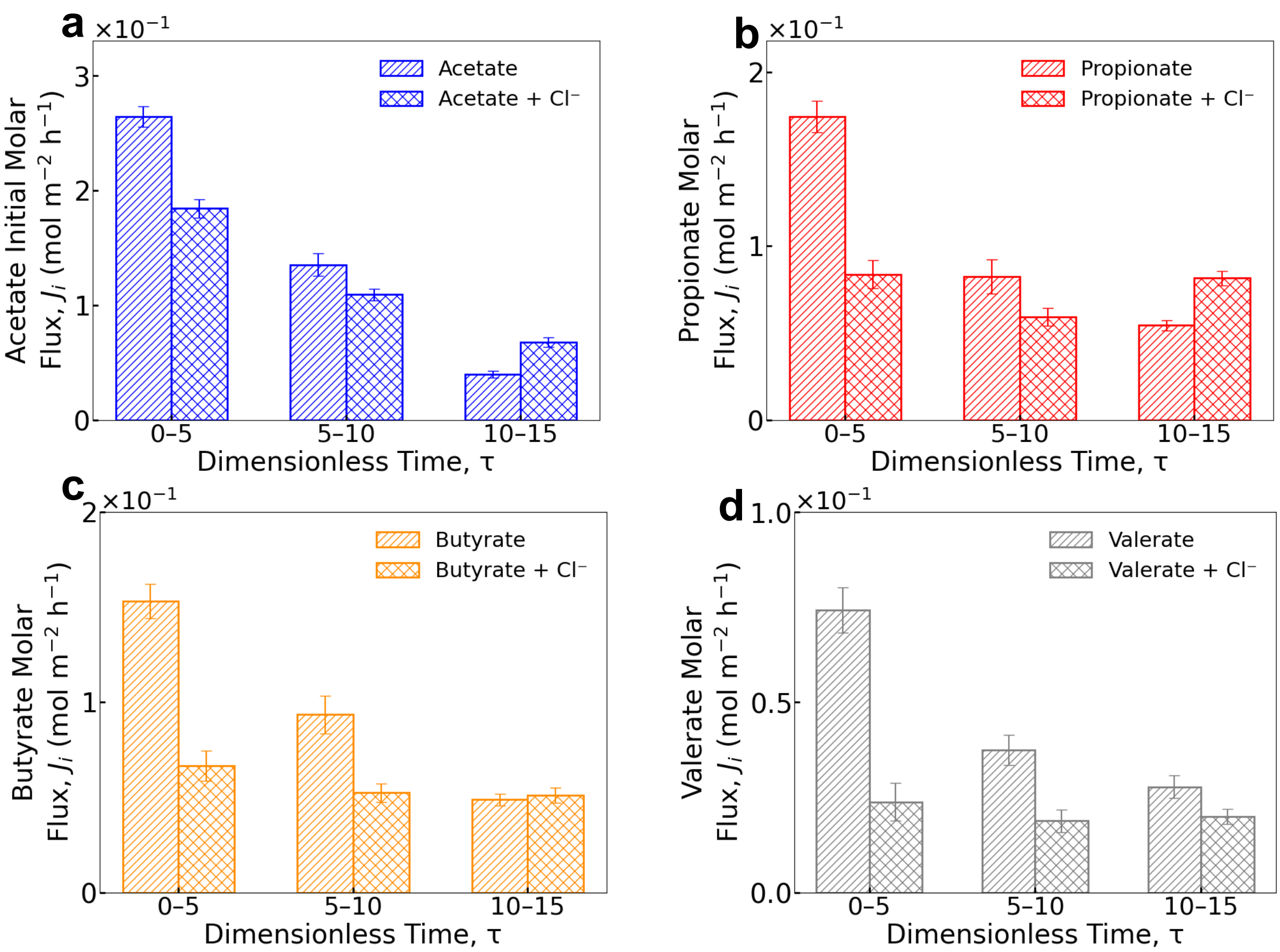

Figure S4: Evolution of VFA molar flux over dimensionless time in the presence and absence of competing chloride ions. Molar flux (J_i) of (a) acetate, (b) propionate, (c) butyrate, and (d) valerate is shown for feed solutions containing only the VFA and for solutions containing both the VFA and Cl^-. Fluxes are averaged over dimensionless time intervals τ = 0–5, 5–10, and 10–15. Error bars represent standard deviations from duplicate experiments.

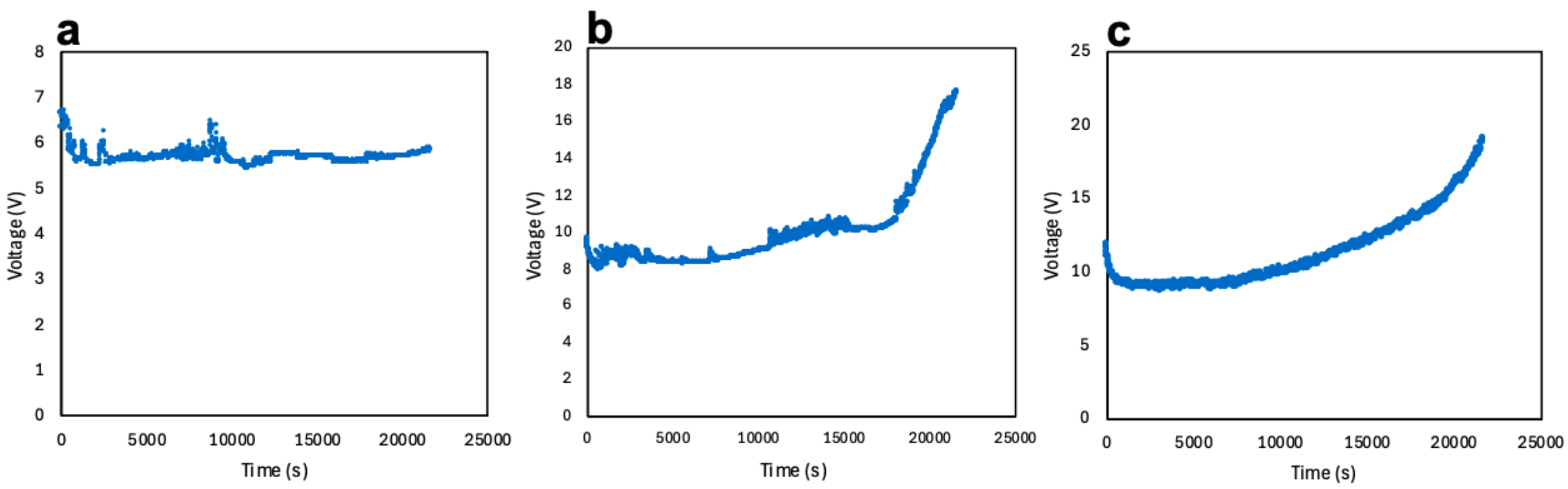

Figure S5: Voltage profile for BPMED experiments at different constant current densities (a) 20 A m^{-2}, (a) 40 A m^{-2}, (a) 60 A m^{-2}.

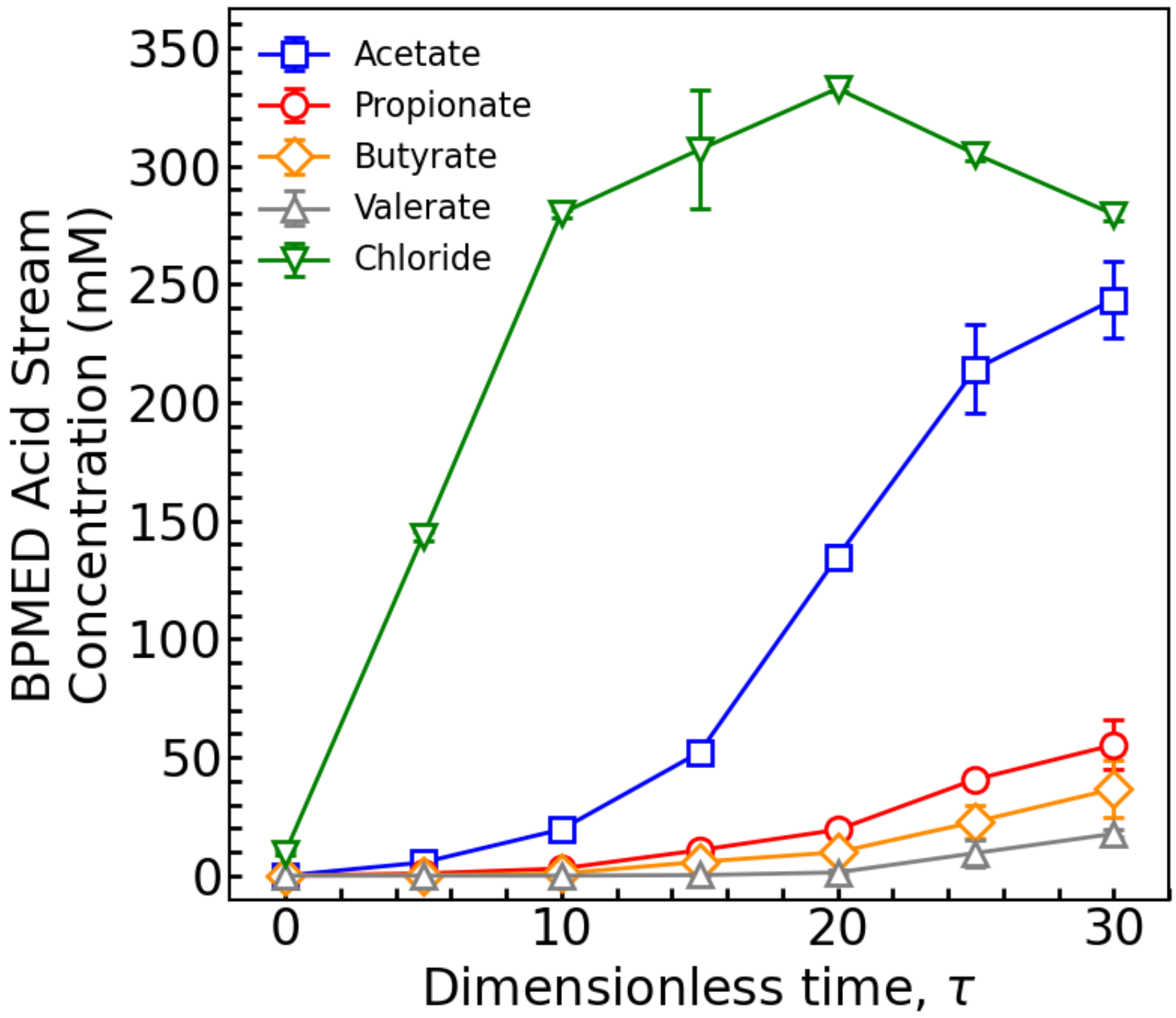

Figure S6: Accumulation of VFAs and chloride in the BPMED acid compartment

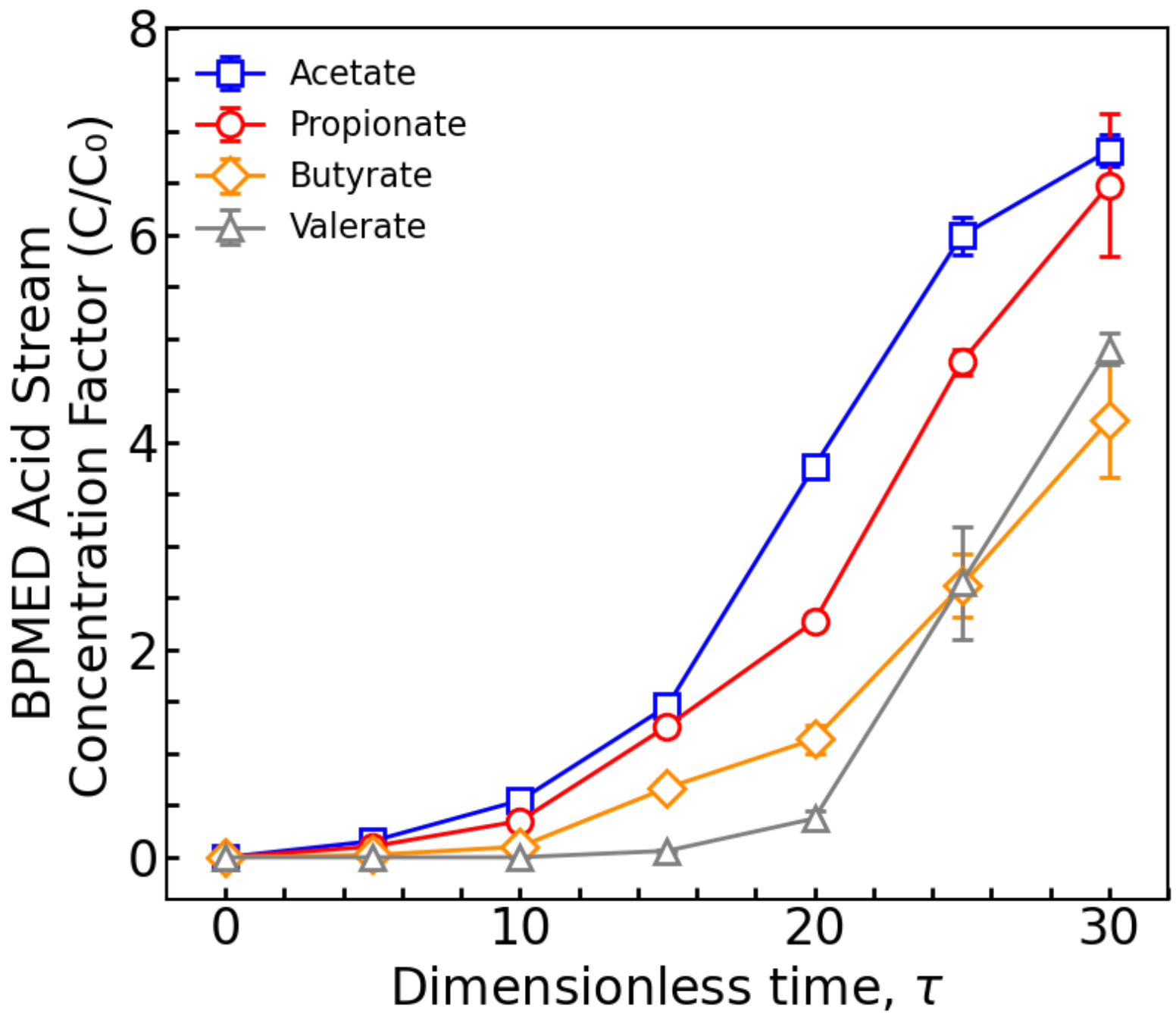

Figure S7: Concentration factor of different VFAs in the BPMED acid compartment

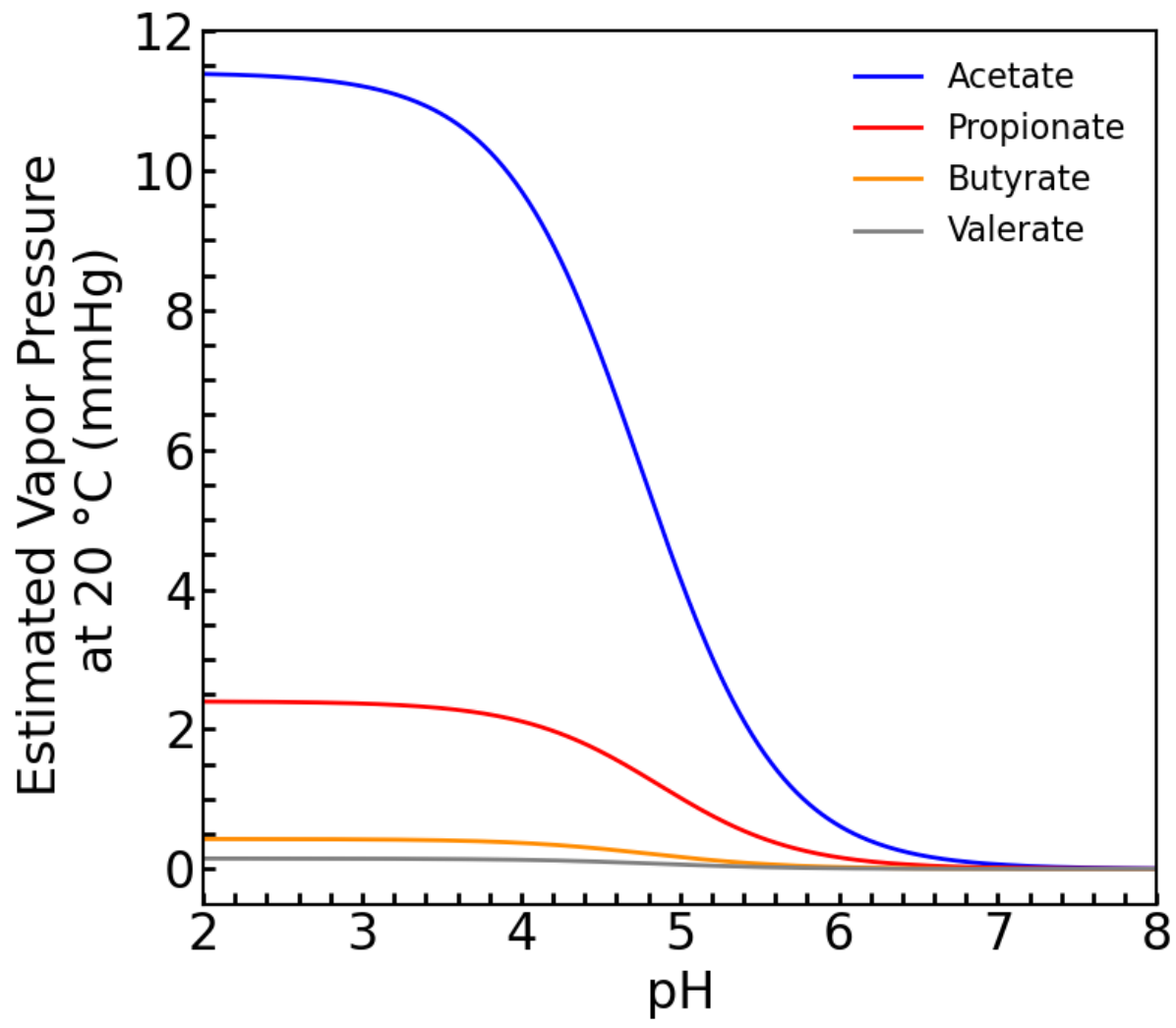

Figure S8: VFA vapor pressures as a function of pH

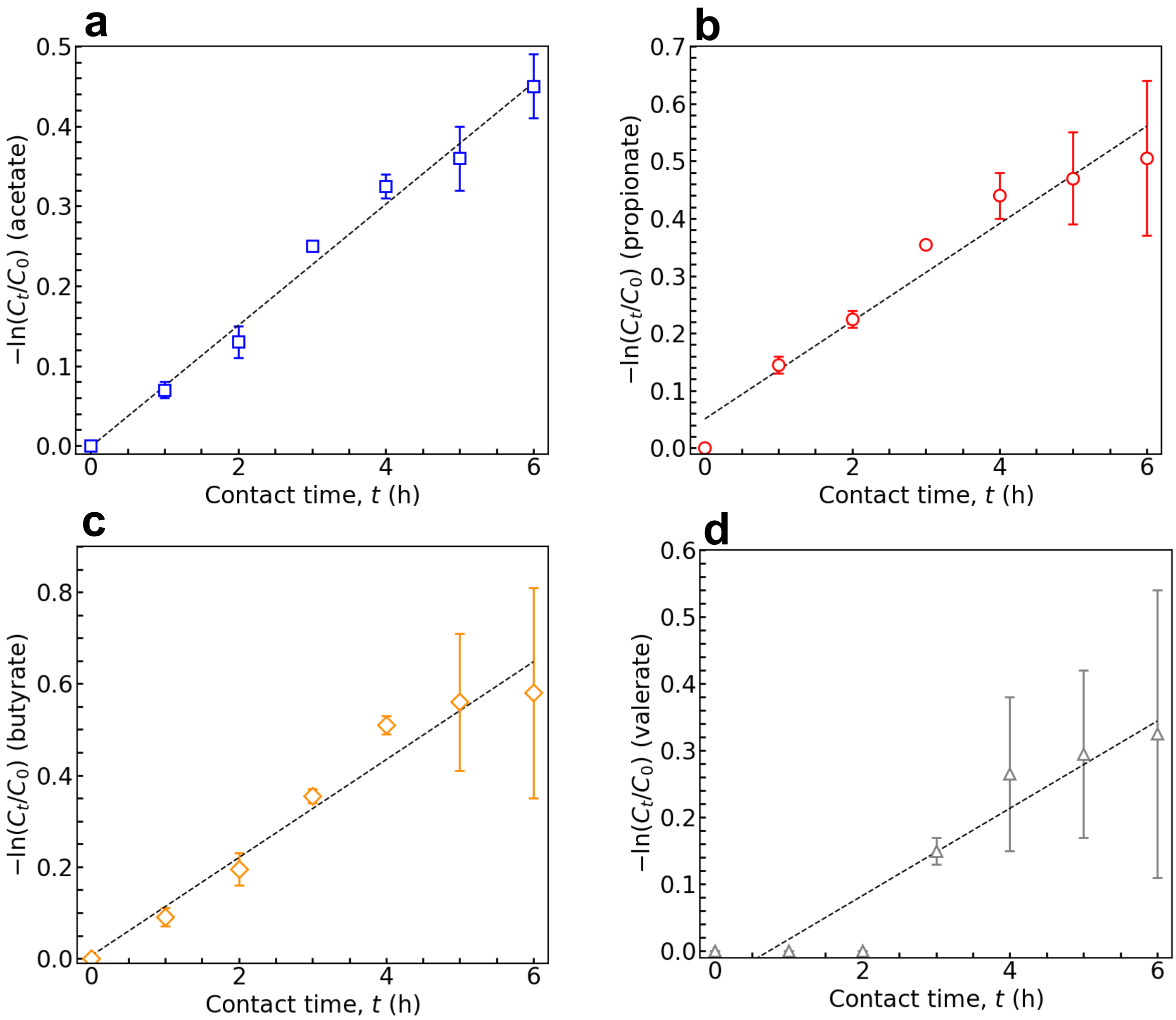

Figure S9: First-order kinetic fits for VFA transport in the HFMC. Linearized plots of $-\ln(C_t/C_0)$ versus contact time for (a) acetate, (b) propionate, (c) butyrate, and (d) valerate during HFMC operation. Error bars denote standard deviations from duplicate experiments. Dashed lines indicate linear regressions used to determine the mass-transfer coefficients.

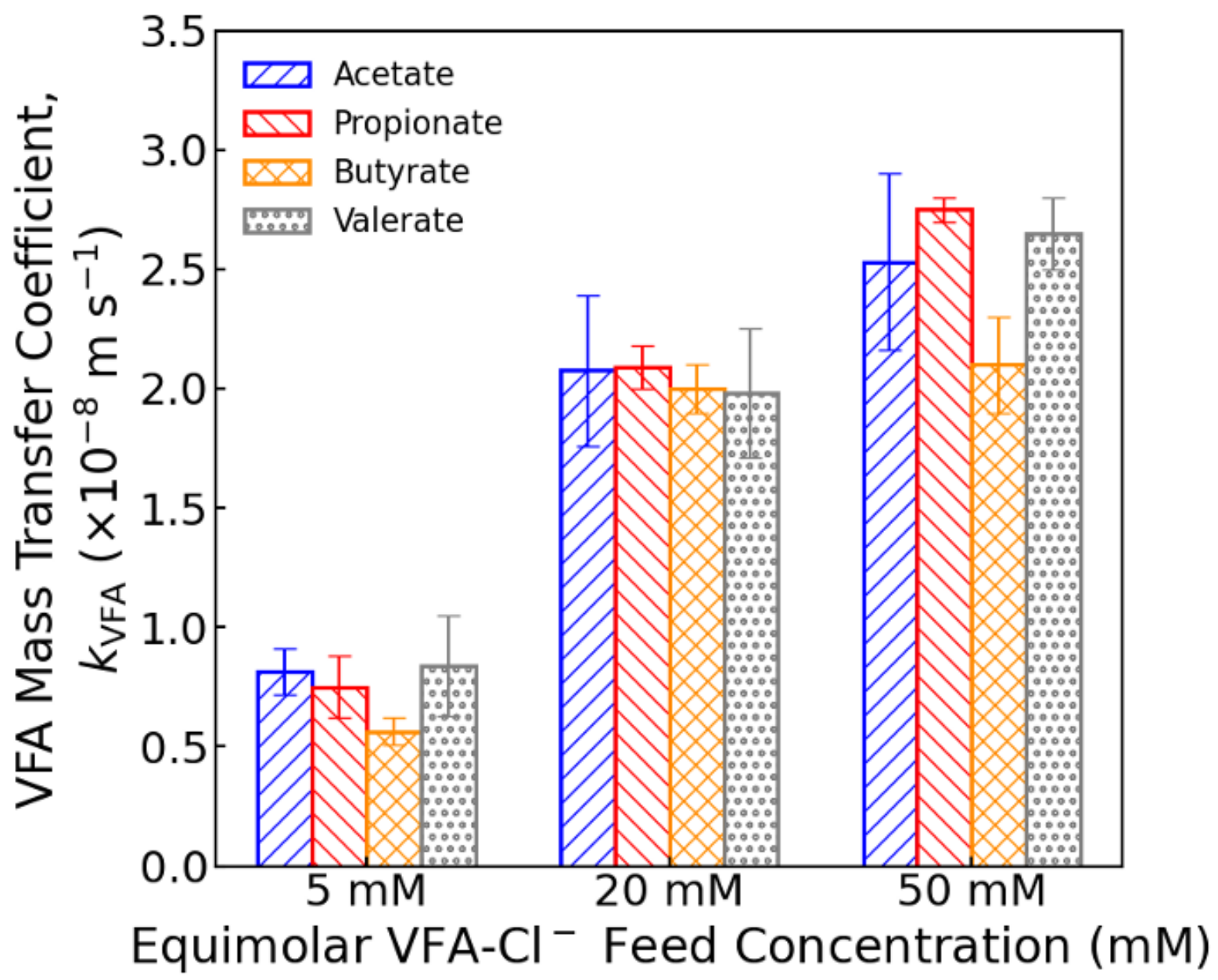

Figure S10: VFA mass transfer coefficients measured in the HFMC for equimolar mixtures of acetate, propionate, butyrate, and valerate at 5, 20, and 50 mM per VFA. No statistically significant differences in the mass transfer coefficients were observed among VFA species at any feed concentration (one-way ANOVA, $p > 0.05$), indicating that HFMC transport exhibits comparatively weak species dependence over the concentration range investigated. Error bars represent standard deviations from triplicate experiments.

Table S7: Percentage recovery of VFA and NH_3 in BPMED and HFMC under different operating conditions.

Condition	% Recovery in BPMED					% Recovery in HFMC				
	Ac	Pr	Bu	Va	NH_3	Ac	Pr	Bu	Va	NH_3
20 A/m^2	55	37	45	0	73	55	41	38	7	87
40 A/m^2	90	93	85	96	100	92	89	85	40	94
60 A/m^2	91	98	84	97	100	92	88	86	49	95
HFMC only control	–	–	–	–	–	68	55	42	29	92

Table S8: Final composition of the recovered VFA and ammonia product streams obtained under different BPMED operating current densities and for the HFMC-only control. Concentrations are reported in the final product streams after completion of the recovery process.

	VFA product stream (mM)				Ammonia product stream (mM)
Condition	Acetate	Propionate	Butyrate	Valerate	NH_4^+
20 A m^{-2}	57	25	0	0	350
40 A m^{-2}	182	49	46	13	554
60 A m^{-2}	430	91	63	24	679
HFMC-only control	93	35	22	0	107

Table S9: Assumptions used for the energy and economic analysis.

Parameter	Value	Unit	Reference
Electricity price	0.0813	$ kWh^{-1}	[4]
Electrochemical HCl & NaOH production energy	0.65	kWh kg^{-1}	[5]
Conventional chemical production energy			
Chlor-alkali NaOH production	2.29	kWh kg^{-1}	[5]
Conventional HCl production	1.38	kWh kg^{-1}	[6]
Chemical price assumptions			
Hydrochloric acid (HCl)	0.23	$ kg^{-1}	[7]
Sodium hydroxide (NaOH)	0.35	$ kg^{-1}	[8]
Product price assumptions			
Ammonium chloride (NH_4Cl)	0.42	$ kg^{-1}	[9]
Acetic acid	1.27	$ kg^{-1}	[10]
Propionic acid	3.83	$ kg^{-1}	[10]
Butyric acid	7.13	$ kg^{-1}	[10]
Valeric acid	3.00	$ kg^{-1}	[10]

References

(1) Annamalai, N.; Elayaraja, S.; Oleskowicz-Popiel, P.; Sivakumar, N.; Al Bahry, S. *Recent developments in bioenergy research*; Elsevier, 2020; pp 237–251.

(2) Goldberg, I.; Rokem, J. S. Organic and fatty acid production, microbial. *Encyclopedia of microbiology* **2009**, 421–442.

(3) Ferby, M.; He, Z. Recovery of both volatile fatty acids and ammonium from simulated wastewater: Performance of membrane contactor and understanding the effects of osmotic distillation. *Journal of Environmental Chemical Engineering* **2024**, *12*, 113760.

(4) U.S. Energy Information Administration Average Price of Electricity to Ultimate Customers by End-Use Sector. https://www.eia.gov/electricity/monthly/epm_table_grapher.php?t=epmt_5_03, 2026; Electric Power Monthly, Table 5.3. Accessed: 2026-01-12.

(5) Kumar, A.; Du, F.; Lienhard, J. H. Caustic soda production, energy efficiency, and electrolyzers. *ACS Energy Letters* **2021**, *6*, 3563–3566.

(6) Boustead, I. Eco-profiles of the European Plastics Industry: Hydrogen Chloride. 2005; http://www.inference.org.uk/sustainable/LCA/elcd/external_docs/hcl_311147f3-fabd-11da-974d-0800200c9a66.pdf, Data last calculated March 2005.

(7) Business Analytiq Hydrochloric Acid Price Index. 2026; https://businessanalytiq.com/procurementanalytics/index/hydrochloric-acid-price-index/, Accessed January 12, 2026.

(8) Business Analytiq Caustic Soda Price Index. 2024; https://businessanalytiq.com/procurementanalytics/index/caustic-soda-price-index/, Accessed January 12, 2026.

(9) IMARC Group Ammonium Chloride Prices, Trend, Chart, Demand, Market Analysis, News, Historical and Forecast Data Report 2026 Edition. 2026; https://www.imarcgroup.com/ammonium-chloride-pricing-report, Accessed January 12, 2026.

(10) Tahmid, M.; Choi, H. J.; Cruz, A. L. C.; Ferreira-Garcia, D.; Abbasi, E.; Botte, G. G.; Hatzell, M. C. Waste-Based Volatile Fatty Acids for Fuel and Chemical Production. *ACS Energy Letters* **2026**,
